# The causal relationships between gut microbiota and venous thromboembolism: a Mendelian randomization study

**DOI:** 10.1186/s41065-025-00389-5

**Published:** 2025-02-20

**Authors:** Pin Huang, Ying Xiao, Ye He

**Affiliations:** 1 Department of Intensive Care Unit (ICU), Sun Yat-sen University Cancer Center, Guangzhou, 510060 P. R. China; 2 Department of Medical Oncology, Sun Yat-sen University Cancer Center, Sun Yat-sen University, Guangzhou, 510060 P. R. China; 3 State Key Laboratory of Oncology in South China, Guangdong Key Laboratory of Nasopharyngeal Carcinoma Diagnosis and Therapy, Guangdong Provincial Clinical Research Center for Cancer, Sun Yat-sen University Cancer Center, Guangzhou, 510060 P. R. China

**Keywords:** Gut microbiota, Venous thromboembolism, Mendelian randomization

## Abstract

**Background:**

Venous thromboembolism (VTE) is still one of the most severe health issues, increasing mortality and lengthening hospital stays. Different abundances of gut microbiota have been clinically linked to VTE and coagulopathy. However, whether gut microbiota affected VTE formation remained uncertain.

**Methods:**

The causative links between VTE and 211 gut microbiota at phylum, class, order, family and genus level were separately investigated using two-sample Mendelian Randomization (MR) analysis. Firstly, single nucleotide polymorphisms (SNPs) locus-wide significantly (*P* < 1.0 × 10^− 5^) related with gut microbiome abundance were extracted from large genome-wide analysis (GWAS) meta-analysis summary data. Instrumental variables (IVs) without pleiotropy were selected using the PhenoScanner and MR PRESSO test. Then, the MR analysis was implemented using the inverse variance weighted (IVW) method. Moreover, weighted median method, MR Egger method, simple median method and MR PRESSO were conducted to validate the causal associations. The reliability of the results was also assessed utilizing various sensitivity analyses, reverse MR analysis and multivariate Mendelian Randomization analysis (MVMR).

**Results:**

We found the phylum Firmicutes was robustly protective against VTE with MR analysis. Moreover, five taxa of Actinobacteria phylum (Bifidobacteriales order, Actinomycetales order, Bifidobacteriaceae family, Actinomycetaceae family, Slackia genus) and two taxa of Firmicutes phylum (Bacillales order, Lachnospiraceae UCG-010 genus) were suggestively protective for VTE. While three taxa of Firmicutes phylum (Bacilli class, Lactobacillales order and Lactococcus genus) might suggestively increase the risk of VTE. Sensitivity analyses indicated no significant horizontal pleiotropy, heterogeneity, or reverse causal associations. Furthermore, MVMR analysis unveiled independently positive causal association of Firmicutes phylum and Lachnospiraceae UCG-010 genus with risk of VTE.

**Conclusion:**

Two taxa of gut microbes (Firmicutes phylum and Lachnospiraceae UCG-010 genus) were independently protective against VTE, which suggests a potential avenue for developing new cost-effective strategies with minor side effects for VTE prevention and treatment.

**Supplementary information:**

The online version contains supplementary material available at 10.1186/s41065-025-00389-5.

## Introduction

Venous thromboembolism (VTE) is a severe medical condition arising from blood clot formation in the veins, which can potentially lead to disability and mortality. Approximately 10 million individuals worldwide suffer from VTE every year. In western regions, over 8% of individuals will be diagnosed with VTE, resulting in approximately 20% deaths [1]. In addition to high incidence and mortality, VTE also imposes a significant economic burden [2], with billions spent on VTE management in the United States [3] and Europe [4] each year. VTE formation is a complex process that mainly involves blood stasis [5], endothelial injury [6], and hypercoagulability [7]. Pregnancy, tumors, diabetes, and smoking have been reported to be associated with increased VTE risk [8, 9]. Thromboprophylaxis is recommended as a standard treatment to prevent VTE, however, the potential for haemorrhage risk is also increased at the same time [10]. Thus, more effective and safe treatment options are needed to be explored.

A comprehensive exploration of the modifiable risk factors is vital for preventing thromboembolism and optimizing thromboprophylaxis usage. The gut microbiota, a community of microorganisms inhabiting the gastrointestinal tract, has emerged as a potential VTE modifiable risk factor. The VTE and control groups have significantly different alpha and beta microbes diversity. The study found that VTE patients exhibited an overgrowth of Blautia, Roseburia, Coprococcus, and Ruminococcus based on small size population (8 VTE patients and 7 healthy controls) [11]. Distinct abundances of gut microbiota were between COVID-19 (Coronavirus disease 2019) patients with coagulopathy and patients without coagulopathy [12]. Microbes from healthy donors increased the thrombinography lag time and downregulated coagulation related plasma proteins levels in subjects with metabolic syndromes [13]. Of note, although microbial alterations have been suggested to be associated with VTE, whether a causal relationship exists between gut microbiota and VTE still remained unclear.

To explore the causal role of exposure on outcome risk, Mendelian Randomization (MR) used instrumental variables (IVs) with genetic variants [14]. Genetic variants are randomly allocated based on random Mendelian genetic variation, minimizing the influence of confounding factors. If the variants significantly associated with an exposure also affect an outcome, it is likely that exposure is causally related to the outcome [15]. Moreover, the abundance of gut microbiota showed significant heritability in the TwinsUK cohort [16], and 13.2% of VTE might be attributed to genetic factors [17]. Thus, it is a feasible approach to use genetic variants to explore the causality between microbial taxa and VTE.

In this study, to assess the causal correction between gut microbiota and VTE, we performed a two sample Mendelian Randomization analysis based on the largest-scale genome wide meta-analysis of VTE and gut microbiota taxa currently. Various sensitivity analyses, reverse MR analysis as well as multivariate Mendelian Randomization analysis (MVMR) were subsequently used to validate our findings.

## Methods

### Data sources

#### Gut microbiota cohorts

As shown in Supplementary Table 1, genome-wide association studies (GWAS) summary data on microbial taxa relative abundance were from the MiBioGen consortium’s GWAS study, comprising 25 cohorts of 18,340 multiethinic subjects, including European mainly, AfroAmerican, Hispanics, East Asian and Middle-East. The MiBioGen consortium’s GWAS study based on the 16SrRNA sequencing profiles of bacterial taxa. The meta-analysis was done with a random effect model if there was heterogeneity [16].

#### VTE cohorts

VTE summary data was extracted from VTE GWAS meta-analysis, including six cohorts of 81,190 European cases with VTE and 1,419,671 European controls. VTE was diagnosed with ICD10 codes. And detailed codes were available in the original meta-analysis supplemental materials [17].

Measurement, quality control and selection of genetic variants were described in the original meta-analysis supplemental materials. The original GWAS studies chosen for this MR analysis have provided all necessary ethical approvals.

### Study design

**Fig. 1:**
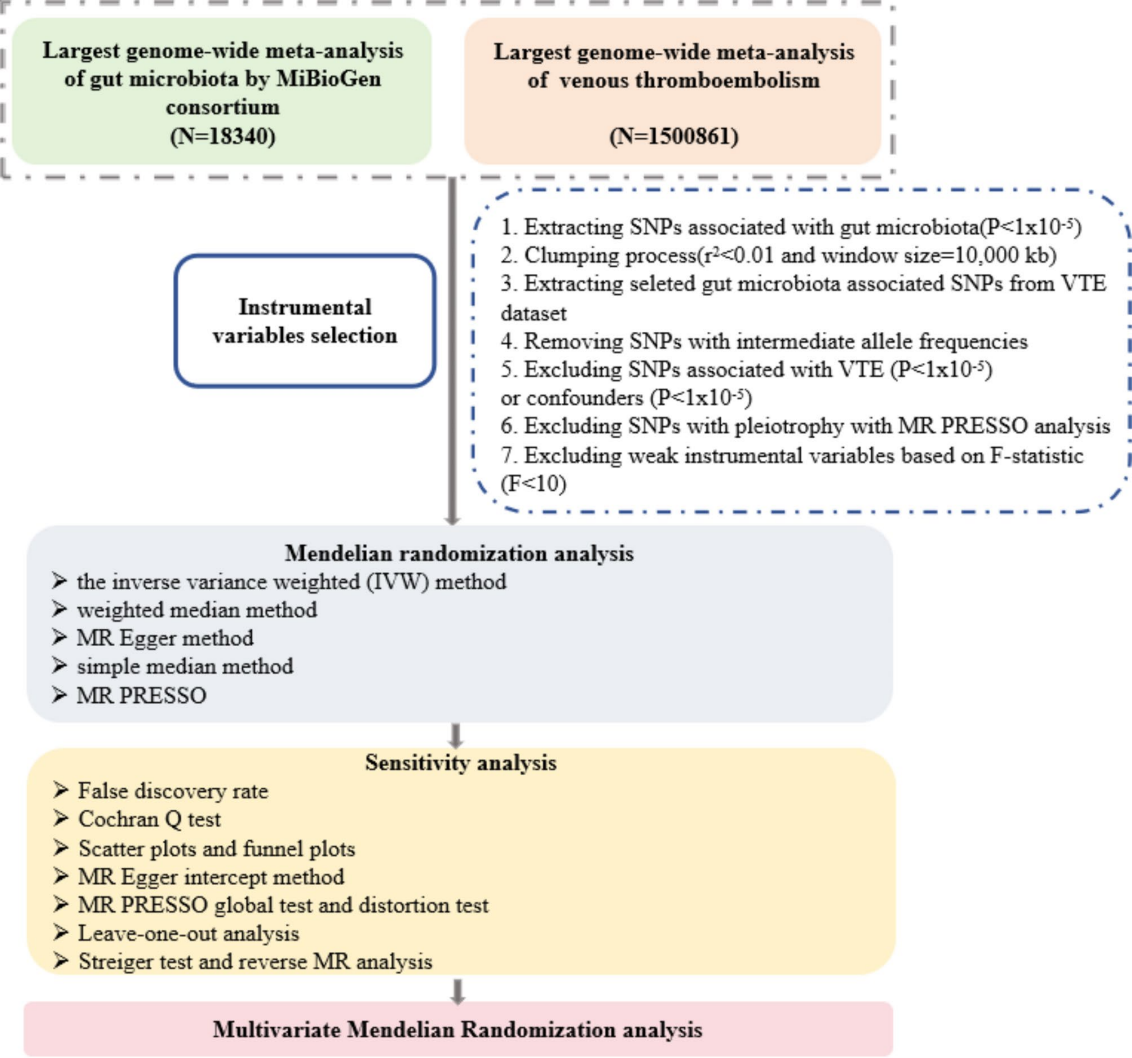
Procedures of MR analysis. SNP: single nucleotide polymorphisms; VTE: venous thromboembolism; MR: mendelian randomization analysis.

The overall designation was shown in Fig. 1. The three necessary assumptions were satisfied in our study: (i) The instrumental variables exhibit a strong correlation with the exposure; (ii) The influence of the IVs on the outcome is only through their impact on the exposure, excluding through any other factors; (iii) The instrumental variables are not associated with any confounders of the outcome [18]. Additionally, the study was guided by STROBE-MR [19] (Supplemental Table 2).

#### Instrumental variables selection

Firstly, the instrumental variables of gut microbiota were extracted following the below criteria:

i. Single nucleotide polymorphisms (SNPs) locus-wide significantly (*P* < 1.0 × 10^− 5^) associated with each bacterial taxon at five taxonomic levels: phylum, class, order, family and genus were selected as potential IVs.
ii. Linkage disequilibrium (LD) between IVs with reference of the 1000 Genomes European Project was analyzed. Only SNPs with r^2^ < 0.01 and window size > 10,000 kb were included to avoid the effects of LD between IVs.
iii. SNPs with intermediate allele frequencies were excluded to avoid ambiguous effects on MR analysis results.
iv. To lower horizontal pleiotropy, IVs strongly correlated (*P* < 1.0 × 10^− 5^) with confounders or VTE were excluded. Smoking was considered a confounder of VTE. The smoking-related SNPs were looked up in Phenoscanner [20]. Then, the MR PRESSO analysis was done to identify IVs associated with any potential confounders. If significant horizontal pleiotropy was detected by the MR PRESSO Global test(*P* < 0.05) and MR PRESSO distortion test(*P* < 0.05), an outlier-corrected MR test was done to investigate the effects of outlier SNPs on analysis. If MR results were significantly changed after excluding the outlier SNPs, then the outlier SNPs would be excluded from the MR analysis.
v. Weak IVs in MR studies could cause bias in MR analysis. F-statistic usually was used to discuss the efficacy estimation of instrumental variables from exposure. F-statistic was calculated according to the formula $\:\text{F}=\left(\frac{\text{n}-\text{k}-1}{\text{k}}\right)\left(\frac{{\text{R}}^{2}}{1-{\text{R}}^{2}}\right)$[21]. n was the sample size of the exposed data, k represented the number of instruments and genetic variants (R^2^) was related to the proportion of variance in the exposure phenotype. An F-statistic less than 10 indicated the presence of weakly predictive instruments.

#### MR analysis and sensitivity analyses

We included SNPs that met al.l the above criteria as IVs for downstream MR analysis. Several sensitivity analyses were carried out to test the reliability of the MR results. We did a reverse MR analysis using the 93 independent significant loci from the GWAS study on VTE [18] as IVs. The reverse MR analysis and streiger test were to verify the causal directions. Furthermore, Leave-one-out analysis, funnel plot and scatter plot were used to assess the heterogeneity of IVs. Furthermore, the Multivariate Mendelian Randomization (MVMR) was conducted to evaluate the intricate interplay among the significant microbes found by two sample MR analysis [22].

### Statistical analysis

The inverse variance weighted (IVW) method was primarily employed for the MR analysis [23]. To control the error rates, the results of multiple tests were corrected with false discovery rate (FDR) for each taxa based on Benjamini-Hochberg (BH) [24]. If FDR less than 0.05, MR analysis was considered as significance. Suggestive significance refers to an MR analysis with P value less than 0.05 and an FDR over 0.05. Weighted median method, MR-Egger, simple median method, Weighted mode and MR-PRESSO were conducted to verify the correlations between bacterial taxa and VTE [25–28].

To validate the reliability and credibility of the results, we carried out a series of sensitivity analyses. Firstly, we quantified heterogeneity with Cochran’s Q statistic; a P value less than 0.05 was considered heterogeneity [23]. All analyses used the IVW random effect model to reduce the effect of heterogeneity. Secondly, we evaluated the SNP bias between variant-specific causal estimates by employing meta-analysis methodologies: scatter plots and funnel plots [29]. Considering that MR Egger may display lower accuracy in specific cases, only both results of MR-Egger regression (*P* < 0.05) and MR-PRESSO method (including global test (*P* < 0.05) and distortion test (*P* < 0.05)) were significant would be considered as horizontal pleiotropy [25, 30]. The outlier-corrected MR test was further conducted to explore the effects of outlier SNPs on the analysis if MR-Egger regression and the MR-PRESSO method showed horizontal pleiotropy. Additionally, we implemented a Leave-one-out analysis on the instrumental variables, through sequentially eliminating each SNP of bacterial taxa. With the remaining SNPs, we recalculated the MR estimates to verify the association between gut microbiota and VTE. If direction of main MR results differed from Leave one out analysis results, the IV excluded in the Leave one out analysis would be considered as an outlier. Multivariate Mendelian Randomization analysis was carried out using weighted regression mode.

All statistical analyses were performed in R4.3.1 using the TwoSampleMR [31].

## Results

### Instrumental variables selection

With gut microbiota as exposure variable, Mendelian Randomization analysis on 14,587 locus-significant SNPs (*P* < 1.0 × 10^− 5^) of 211 gut microbiota, including nine phyla, 16 classes, 20 orders, 35 families, and 131 genera, was carried out. Through the clumping process, 2877 SNPs were included. Eleven SNPs strongly associated with VTE directly (*P* < 1.0 × 10^− 5^), 422 SNPs with intermediate frequency allele and 116 SNPs unavailable in VTE data were excluded. 2328 SNPs passed through instrumental variables selection criteria (detailed SNPs as seen in Supplemental Table 3).

While VTE was exposure variable, 93 independent, significant SNPs (*P* < 5.0 × 10^− 8^) from VTE GWAS meta-analysis were extracted. rs4002471 was excluded because of directly associated with Ruminococcustorquesgroup id.14,377. So 92 loci were used as instrumental variables (detailed SNPs as seen in Supplemental Table 4).

Supplemental Tables 3–4 showed that no weak IV was excluded (F statistics of all including IVs > 10). MR PRESSO test combined global test (*P* = 0.039) and distortion test (*P* = 0.015) results, only class Lentisphaeria id.2250 had outlier SNP. However, outlier SNP did not significantly affect the MR results after excluding outlier SNPs (P_all = 0.43, P_excluding outliers = 0.97). Thus, there were no IVs with significant horizontal pleiotropy based on MR PRESSO analysis (Supplemental Table 5).

### Causal relationships between gut microbiota and VTE risk

#### Results of MR analysis and sensitivity analysis

**Fig. 2:**
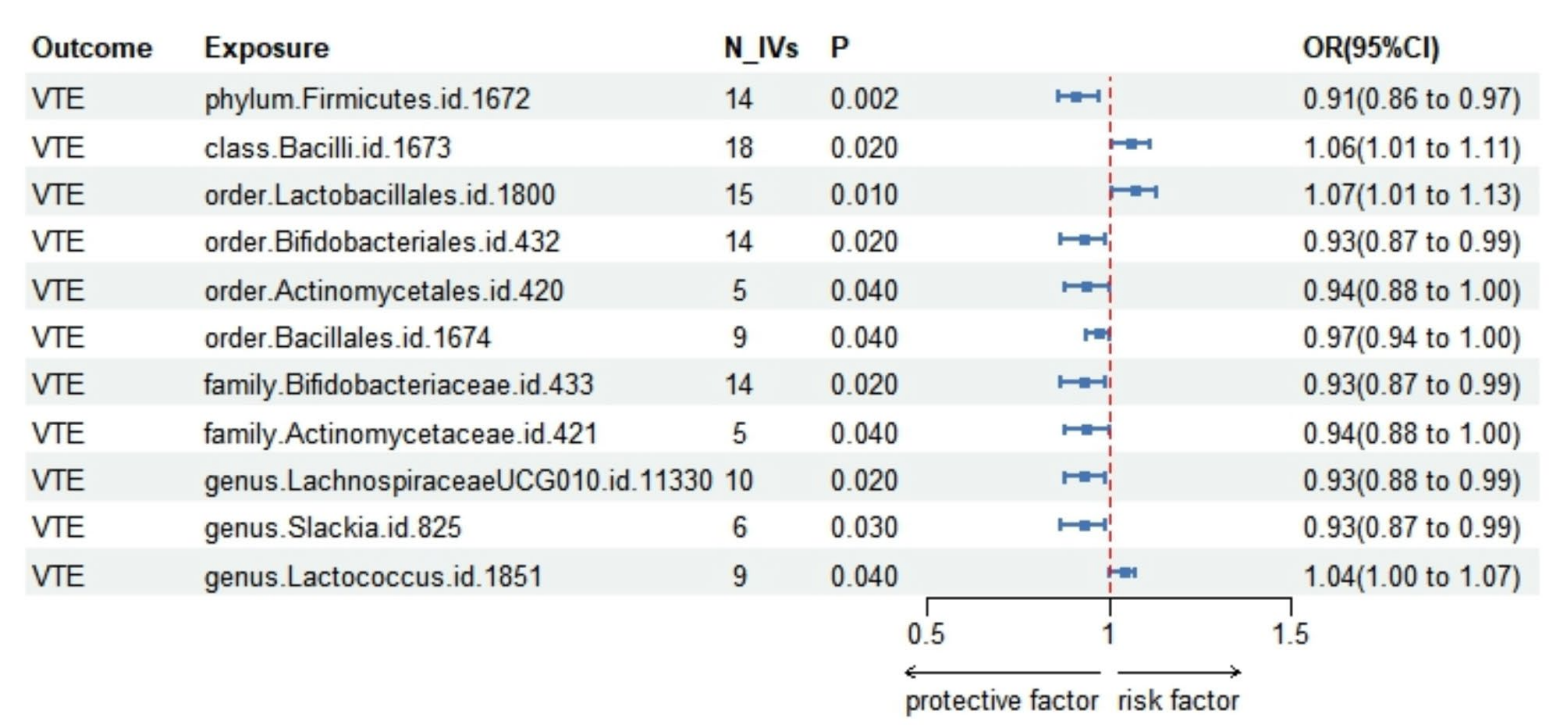
Mendelian randomization results of causal associations between gut microbiota and VTE risk. VTE: venous thromboembolism; N_IVs: numbers of instrumental variable; P: probability; OR: odds ratio; CI: confidence interval

**Fig. 3:**
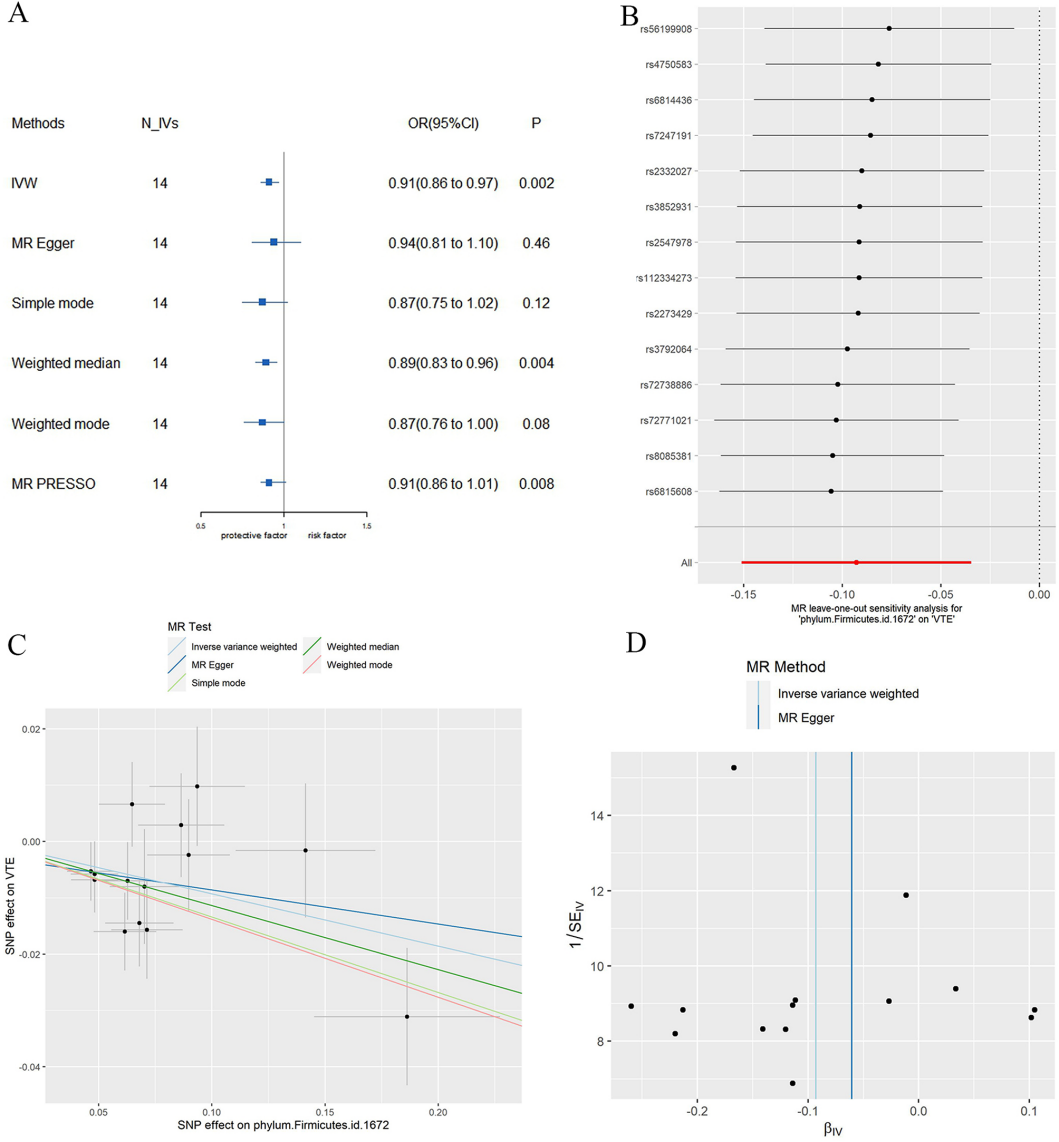
MR results between phylum Firmicutes and VTE. (**A**) Forest plot of MR results between phylum Firmicutes and VTE using the inverse variance weighted, MR Egger, simple mode, weighted median, weighted model methods and MR PRESSO respectively; (**B**) Leave-one out test plot of the causal effect of phylum Firmicutes on VTE risk. The results was recalculated through excluding one SNP per time. (**C**) Scatter plot of the causal effect of phylum Firmicutes on VTE risk; (**D**) Funnel plot of the causal effect of phylum Firmicutes on VTE risk

As shown in Fig. 2; Table 1, Firmicutes phylum id.1672 was significantly negatively associated with VTE (OR(95%CI) = 0.91(0.86, 0.97), *P* = 0.002, FDR = 0.02). The direction of MR analysis results using the weighted median method, MR-Egger, simple median method, weighted mode and MR PRESSO were consistent with using the IVW method (Fig. 3A; Table 1). Heterogeneity for gut microbiome-VTE causal associations was not observed (P Cochran’s Q_IVW = 0.33). As shown in Table 2, neither the Egger Intercept test (*P* = 0.67) nor the MR-PRESSO Global test (*P* = 0.37) recognized significant horizontal pleiotropy. Similarly, the MR-PRESSO distortion test and outlier test did not identify any outlier SNPs that could lead to horizontal pleiotropy. Leave-one-out analysis for Firmicutes phylum id.1672 showed each including SNPs had the consistent effect in Fig. 3B. The scatter plot and funnel plot showed no obvious outlier (Fig. 3C and 3D).

**Table 1:** Significant MR results of causal associations between gut microbiota and VTE risk

Taxa(Exposure)	Method	N_IVs	OR (95%CI)	*P*	FDR	Q_pval
**phylum.Firmicutes.id.1672**	IVW	14	0.91 (0.86,0.97)	0.002	**0.02**	0.33
	MR Egger	14	0.94 (0.81,1.1)	0.46		0.28
	Simple mode	14	0.87 (0.75,1.02)	0.12		
	Weighted median	14	0.89 (0.83,0.96)	0.004		
	Weighted mode	14	0.87 (0.76,1)	0.08		
	MR PRESSO	14	0.91 (0.86,1.01)	0.008		
class.Bacilli.id.1673	IVW	18	1.06 (1.01,1.11)	0.02	0.35	0.55
	MR Egger	18	0.98 (0.86,1.12)	0.77		0.59
	Simple mode	18	1.03 (0.92,1.15)	0.65		
	Weighted median	18	1.03 (0.97,1.1)	0.37		
	Weighted mode	18	1.02 (0.93,1.12)	0.67		
	MR PRESSO	18	1.06 (1.01,0.97)	0.03		
order.Actinomycetales.id.420	IVW	5	0.94 (0.88,1)	0.04	0.19	0.86
	MR Egger	5	0.99 (0.86,1.14)	0.89		0.87
	Simple mode	5	0.94 (0.85,1.04)	0.29		
	Weighted median	5	0.94 (0.88,1.02)	0.12		
	Weighted mode	5	0.96 (0.88,1.05)	0.39		
	MR PRESSO	5	0.94 (0.91,0.97)	0.02		
order.Bacillales.id.1674	IVW	9	0.97 (0.94,1)	0.04	0.19	0.86
	MR Egger	9	0.89 (0.77,1.03)	0.17		0.91
	Simple mode	9	0.99 (0.93,1.06)	0.80		
	Weighted median	9	0.98 (0.94,1.02)	0.23		
	Weighted mode	9	0.99 (0.93,1.06)	0.82		
	MR PRESSO	9	0.97 (0.95,0.98)	0.02		
order.Bifidobacteriales.id.432	IVW	14	0.93 (0.87,0.99)	0.02	0.19	0.09
	MR Egger	14	0.78 (0.62,0.97)	0.05		0.16
	Simple mode	14	0.94 (0.82,1.09)	0.43		
	Weighted median	14	0.93 (0.87,1.01)	0.07		
	Weighted mode	14	0.94 (0.79,1.12)	0.50		
	MR PRESSO	14	0.93 (0.87,0.96)	0.04		
order.Lactobacillales.id.1800	IVW	15	1.07 (1.01,1.13)	0.01	0.19	0.51
	MR Egger	15	1.02 (0.9,1.17)	0.73		0.47
	Simple mode	15	1.02 (0.9,1.15)	0.77		
	Weighted median	15	1.02 (0.95,1.1)	0.60		
	Weighted mode	15	1.02 (0.92,1.12)	0.74		
	MR PRESSO	15	1.07 (1.02,0.98)	0.02		
family.Actinomycetaceae.id.421	IVW	5	0.94 (0.88,1)	0.04	0.65	0.86
	MR Egger	5	0.99 (0.86,1.14)	0.89		0.87
	Simple mode	5	0.94 (0.85,1.05)	0.32		
	Weighted median	5	0.94 (0.87,1.02)	0.14		
	Weighted mode	5	0.96 (0.88,1.05)	0.42		
	MR PRESSO	5	0.94 (0.91,0.97)	0.02		
family.Bifidobacteriaceae.id.433	IVW	14	0.93 (0.87,0.99)	0.02	0.65	0.09
	MR Egger	14	0.78 (0.62,0.97)	0.05		0.16
	Simple mode	14	0.94 (0.82,1.09)	0.44		
	Weighted median	14	0.93 (0.87,1.01)	0.08		
	Weighted mode	14	0.94 (0.78,1.13)	0.53		
	MR PRESSO	14	0.93 (0.87,0.96)	0.04		
genus.LachnospiraceaeUCG010.id.11,330	IVW	10	0.93 (0.88,0.99)	0.02	0.95	0.82
	MR Egger	10	0.9 (0.78,1.04)	0.20		0.86
	Simple mode	10	0.92 (0.82,1.03)	0.18		
	Weighted median	10	0.93 (0.86,1)	0.04		
	Weighted mode	10	0.92 (0.82,1.03)	0.17		
	MR PRESSO	10	0.93 (0.9,1)	0.01		
genus.Lactococcus.id.1851	IVW	9	1.04 (1,1.07)	0.04	0.95	0.50
	MR Egger	9	1 (0.85,1.16)	0.97		0.58
	Simple mode	9	1.07 (0.99,1.15)	0.14		
	Weighted median	9	1.05 (1,1.1)	0.05		
	Weighted mode	9	1.06 (0.99,1.15)	0.15		
	MR PRESSO	9	1.04 (1,0.91)	0.05		
genus.Slackia.id.825	IVW	6	0.93 (0.87,0.99)	0.03	0.95	0.13
	MR Egger	6	0.81 (0.52,1.26)	0.40		0.16
	Simple mode	6	0.98 (0.89,1.09)	0.74		
	Weighted median	6	0.97 (0.9,1.04)	0.37		
	Weighted mode	6	0.98 (0.88,1.09)	0.72		
	MR PRESSO	6	0.93 (0.87,0.88)	0.08		

MR: Mendelian Randomization analysis; OR: odds ratio; CI: confidence interval; IVW: Inverse Variance Weighted; N_IVs: numbers of instrumental variable; P: probability; FDR: false discovery rate; Q_pval: p value calculated by Cochran’s Q statistic.

**Table 2:** Pleiotropy tests for MR analysis

Exposure	P_Egger	P_global	P_distortion	P_outiercorrected	Dir_steiger	P_steiger
phylum.Firmicutes.id.1672	0.67	0.37	NA	NA	TRUE	7.64E-89
class.Bacilli.id.1673	0.24	0.58	NA	NA	TRUE	1.69E-114
order.Actinomycetales.id.420	0.49	0.83	NA	NA	TRUE	8.66E-27
order.Bacillales.id.1674	0.30	0.87	NA	NA	TRUE	1.61E-47
order.Bifidobacteriales.id.432	0.13	0.07	NA	NA	TRUE	5.84E-102
order.Lactobacillales.id.1800	0.51	0.54	NA	NA	TRUE	5.57E-102
family.Actinomycetaceae.id.421	0.49	0.85	NA	NA	TRUE	7.46E-27
family.Bifidobacteriaceae.id.433	0.13	0.07	NA	NA	TRUE	5.84E-102
genus.LachnospiraceaeUCG010.id.11,330	0.61	0.89	NA	NA	TRUE	8.77E-62
genus.Lactococcus.id.1851	0.62	0.61	NA	NA	TRUE	2.36E-57
genus.Slackia.id.825	0.57	0.20	NA	NA	TRUE	3.10E-38

MR: Mendelian Randomization analysis; P_Egger: P value was calculated using MR Egger regression; P_global, P_distortion, P_outiercorrected represent P value calculated by MR PRESSO method; Dir_steiger means direction of MR results; P_steiger: P value from streiger test.

According to our results, Bacilli class id.1673 (OR(95%CI) = 1.06(1.01, 1.11), *P* = 0.02, FDR = 0.35), Lactobacillales order id.1800 (OR(95%CI) = 1.07(1.01, 1.13), *P* = 0.01, FDR = 0.19), and Lactococcus genus id.1851 (OR(95%CI) = 1.04(1.00, 1.07), *P* = 0.04, FDR = 0.95) might suggestive significant increased the risk of VTE. While Bifidobacteriales order id.432 (OR(95%CI) = 0.93(0.87, 0.99), *P* = 0.02, FDR = 0.19), Actinomycetales order id.420 (OR(95%CI) = 0.94(0.88, 1.00), *P* = 0.04, FDR = 0.19), Bacillales order id.1674 (OR(95%CI) = 0.97(0.94, 1.00), *P* = 0.04, FDR = 0.19), Bifidobacteriaceae family id.433 (OR(95%CI) = 0.93(0.87, 0.99), *P* = 0.02, FDR = 0.65), Actinomycetaceae family id.421 (OR(95%CI) = 0.94(0.88, 1.00), *P* = 0.04, FDR = 0.65), LachnospiraceaeUCG-010 genus id.11,330 (OR(95%CI) = 0.93(0.88, 0.99), *P* = 0.02, FDR = 0.95), and Slackia genus id.825 (OR(95%CI) = 0.93(0.87, 0.99), *P* = 0.03, FDR = 0.95) might play a suggestive significantly protective value in subjects with VTE.

For the suggestive significant taxa of bacteria, mostly the directions of MR analysis results using the weighted median method, MR-Egger, simple median method, weighted mode and MR PRESSO were similar to the results using IVW method, except for the MR-Egger test of Bacilli class id.1673 (OR(95%CI) = 0.98 (0.86,1.12), *P* = 0.77) differed from IVW method (OR(95%CI) = 1.06(1.01, 1.11), *P* = 0.02) (Tables 1and Supplemental Fig. 1B-10B). Additional Leave-one-out analyses, scatter plots and funnel plots were shown in Supplemental Figs. 1–10. Furthermore, according to the Leave-one-out analysis of each taxa, their single SNP was all consistent with the overall MR analysis. And funnel plots for the IVW method including IVs for each taxon were nearly symmetrical except in Actinomycetales order id.420, Actinomycetaceae family id.421 and Slackia genus id.825 because of the small number of IVs (N_IVs = 5).

#### Reverse causal associations between gut microbiota and VTE risk

Streiger test showed directions of the eleven significant causal associations were from bacteria to VTE (Table 2) (all P_streiger < 0.05). And as shown in Table 3, reverse MR analysis using the IVW method did not detect any significant causal effects of VTE on gut microbiota (all *P* > 0.05). There was no heterogeneity observed in reverse MR analysis (all Cochran’s Q statistic *P* > 0.05).

**Table 3:** Bidirectional causal effects between gut microbiota and VTE risk

Taxa(outcome)	Method	N_IVs	OR(95%CI)	*P*	Q_pval	FDR
phylum.Firmicutes.id.1672	IVW	76	0.99 (0.95,1.04)	0.65	0.19	0.95
class.Bacilli.id.1673	IVW	76	1 (0.96,1.05)	0.83	0.80	0.86
order.Actinomycetales.id.420	IVW	75	1.01 (0.95,1.08)	0.65	0.63	1.00
order.Bacillales.id.1674	IVW	69	1.02 (0.92,1.13)	0.75	0.64	1.00
order.Bifidobacteriales.id.432	IVW	76	0.99 (0.94,1.04)	0.60	0.44	1.00
order.Lactobacillales.id.1800	IVW	76	1 (0.96,1.05)	0.98	0.87	1.00
family.Actinomycetaceae.id.421	IVW	75	1.01 (0.95,1.08)	0.66	0.62	1.00
family.Bifidobacteriaceae.id.433	IVW	76	0.99 (0.94,1.04)	0.60	0.44	1.00
genus.LachnospiraceaeUCG010.id.11,330	IVW	76	0.97 (0.92,1.03)	0.36	0.06	0.94
genus.Lactococcus.id.1851	IVW	73	0.91 (0.83,1.01)	0.05	0.51	0.93
genus.Ruminococcustorquesgroup.id.14,377	IVW	76	0.98 (0.94,1.03)	0.48	0.85	0.94
genus.Slackia.id.825	IVW	75	0.98 (0.91,1.06)	0.64	0.78	0.94

VTE: venous thromboembolism; OR: odds ratio; CI: confidence interval; IVW: Inverse Variance Weighted; N_IVs: numbers of instrumental variable; P: probability; FDR: false discovery rate; Q_pval: p value calculated by Cochran’s Q statistic;

#### Results of multivariate mendelian randomization analysis

To unveil the most influential microbes on VTE, MVMR was conducted. We adjusted all microbes strongly associated with VTE in MVMR, including Firmicutes phylum id.1672, Bacilli class id.1673, Actinomycetales order id.420, Bifidobacteriales order id.432, Lactobacillales order id.1800, Actinomycetaceae family id.421, Bifidobacteriaceae family id.433, Lachnospiraceae UCG-010 genus id.11,330, Lactococcus genus id.1851, Slackia genus id.825. Only phylum Firmicutes id.1672 (beta=-0.081, *P* = 0.011) and Lachnospiraceae UCG-010 genus id.11,330(beta=-0.076, *P* = 0.011) showed significant decreased risk of VTE. The other taxa of gut microbiota might affect on VTE through Firmicutes phylum or Lachnospiraceae UCG-010 genus (Table 4).

**Table 4:** Results of multivariate mendelian randomization analysis

Taxa (Exposure)	Outcome	N_IVs	Beta	SE	*P*-value
**phylum.Firmicutes.id.1672**	VTE	70	-0.081	0.032	**0.011**
class.Bacilli.id.1673	VTE	70	-0.001	0.028	0.973
order.Actinomycetales.id.420	VTE	70	-0.001	0.030	0.982
order.Bacillales.id.1674	VTE	69	-0.026	0.013	0.052
order.Bifidobacteriales.id.432	VTE	70	0.000	0.024	1.000
order.Lactobacillales.id.1800	VTE	70	0.001	0.029	0.965
family.Actinomycetaceae.id.421	VTE	67	0.001	0.030	0.983
family.Bifidobacteriaceae.id.433	VTE	70	0.000	0.024	1.000
**genus.LachnospiraceaeUCG010.id.11,330**	VTE	69	-0.076	0.030	**0.011**
genus.Lactococcus.id.1851	VTE	68	0.027	0.016	0.083
genus.Slackia.id.825	VTE	70	-0.010	0.025	0.691

N_IVs: numbers of instrumental variable; VTE: venous thromboembolism; SE: standard error;

## Discussion

We first demonstrated the strong negative causal relationships between Firmicutes phylum and VTE. Multiple sensitivity analyses confirmed the consistency with Firmicutes phylum in the MR analysis. Moreover, five taxa of Actinobacteria phylum (Bifidobacteriales order, Actinomycetales order, Bifidobacteriaceae family, Actinomycetaceae family, Slackia genus) and two taxa of Firmicutes phylum (Bacillales order, Lachnospiraceae UCG-010 genus) were suggestively protective for VTE. While three taxa of Firmicutes phylum (Bacilli class, Lactobacillales order and Lactococcus genus) might suggestively increased the risk of VTE. And the sensitivity analysis were almost consistent with the MR results. Finally, phylum Firmicutes or genus Lachnospiraceae UCG-010 were independently decreased risk of VTE after adjusting other microbiota using MVMR.

Recent studies have shown a significant clinical associations between the gut microbiota and the risk of thrombosis formation without focusing on any particular taxa [12, 13]. Yang et al. did similar MR analysis to study the association between VTE and 74 taxa of gut bacteria. They have shown a positive causal genetic relationship between Streptococcaceae and DVT using IVW (OR = 1.003, *P* = 0.04) [32]. Our study population included their cohorts. Moreover, Yang’s results would not be significant if the analysis was corrected for multiple times tests. And our results for Streptococcaceae (OR = 1.04) got the same direction with Yang et al.

Our findings have shown that Firmicutes phylum and Lachnospiraceae UCG-010 genus played independently protective roles against VTE. And Lachnospiraceae UCG-010 genus affiliates to Firmicutes phylum. Firmicutes is one of the most abundant phylum in the human gut microbiome. It is involved in human metabolic and immune health, yet the specific role of Firmicutes in VTE is still being investigated. Many species of Firmicutes might influence blood coagulation by producing specific metabolites. For example, certain taxa of Firmicutes bacteria such as Bacillus subtilis can produce Menaquinone, a form of Vitamin K2 [33], which plays a crucial role in blood coagulation, and the synthesis of several coagulation factors such as prothrombin and clotting factors VII, IX, and X. Other taxa of phylum Firmicutes were associated with the production of short-chain fatty acids (SCFAs), primarily composed of acetate, butyrate and propionate. The genera Clostridium and Ruminococcus could synthesize the acetate through Wood-Ljungdahl pathway or via acetyl-CoA. Butyrate, a major component of SCFAs, could be produced by various species of Coprococcus catus, Eubacterium rectale and Eubacterium hallii. Propionate was produced from Ruminococcus obeum [34]. Lachnospiraceae was also positive associated with level of short-chain fatty acid content. Furthermore, mice with low abundance of Lachnospiraceae had impaired blood-brain barrier integrity and a proinflammatory condition [35]. Some studies suggested that SCFAs might influence thrombus formation by regulating immune and inflammatory responses. Butyrate could inhibit inflammatory responses and maintain the stability of the vascular endothelium, which reduced thrombus formation [36]. Another study pointed out that butyrate and propionate could regulate platelet function, which might impact thrombus formation [37].

We also concluded five taxa of Actinobacteria phylum (Bifidobacteriales order, Actinomycetales order, Bifidobacteriaceae family, Actinomycetaceae family, Slackia genus) might protect against VTE. Bifidobacterium has been found to lower cholesterol levels in the blood, with potential mechanisms including reduced cholesterol absorption, increased cholesterol excretion, prevented intestinal infections, and might fight against inflammatory bowel diseases [38]. Moreover, the gut microbiota might reduce the risk of systemic inflammation and thrombosis formation by maintaining intestinal barrier function and preventing endogenous toxins from entering the bloodstream [39].

Rare studies focused on the treatment for microbes alterations in VTE patients. However, probiotics increased instestinal barrier integrity and reduced inflammation in cardiovascular disease [40]. Firmicutes and its metabolites, as mentioned above, SCFAs, Menaquinone, show broad clinical application prospects in treating various diseases. For example, available Firmicutes phylum drugs (SER109), is approved for recurrent Clostridioides difficile infection [41]. And SCFAs have shown the effect of prevention of cardiovascular disease [42]. The relative abundance of Lachnospiraceae was inversely correlated with blood pressure and lipid profiles [43]. Also, the utilization of probiotics in clinical practice was recommended for gastrointestinal diseases, such as inflammatory bowel disease [44], constipation and diarrhea [45]. Dietary changes [46, 47], probiotics supplement, and fecal microbiota transplant [48] would be optional easy available choice to change microorganism compositions. In future, more studies are needed to explore the role of beneficial microbes supplementation in VTE prevention.

Our study analyzed genetic causality between microbes and VTE using SNPs from GWAS summary data with a large sample size. MR results has a strong genetic causal inference ability, without obvious horizontal pleiotropy and heterogeneity, which enhances the reliability of our results. Nevertheless there were certain limitations in this study. Firstly, due to the limitation of significant gut microbiota loci numbers, the IVs in MR analysis were at locus wide significant level which would increase false negative rate. Sensitivity analysis and heterogeneity analyses were unable to be conducted using the small number of IVs, which were unable to assess the reliability of results. Thus we did MR analysis based on locus wide significant SNPs of gut microbiome. Secondly, since the smallest taxonomic level of the gut microbiota dataset in the MR analyses was genus, the species of microbes effects need to be explored in future. Then, individuals included in this study were mainly European, and the other ethnic populations were warranted to be explored. Lastly, our study carried out MR analysis to verify the causal relationship in Firmicutes phylum and Lachnospiraceae UCG-010 genus, further validation through experimental and clinical studies would be need for Firmicutes drugs application on preventing VTE.

## Conclusions

Utilizing the largest GWAS summary data, we assessed the causal relationship between VTE and the gut microbiota, and discovered that Firmicutes phylum and Lachnospiraceae UCG-010 genus might play independently protective roles against VTE. Our findings provide additional evidence for the causality relationship between these two taxa of bacteria and VTE. Additional studies are warranted to investigate the potential mechanisms explaining for the two taxa in the pathogenesis of VTE, and to validate their roles in VTE prevention as novel therapeutic targets in clinical practice.

## Electronic supplementary material

Below is the link to the electronic supplementary material.

Supplementary Material 1

Supplementary Material 2

## Data Availability

VTE GWAS data could be download from https://www.decode.com/summarydata, and gut microbiota summary data could be obtained from www.mibiogen.org.
