# Supplementary material for: The causal relationships between gut microbiota and venous thromboembolism: a Mendelian randomization study
Source: Hereditas. 2025 Feb 20;162:25. doi: 10.1186/s41065-025-00389-5 (PMC11841150; doi:10.1186/s41065-025-00389-5)
Supplement: Supplementary file 2 — Supplementary Material 2 [file 41065_2025_389_MOESM2_ESM.docx]

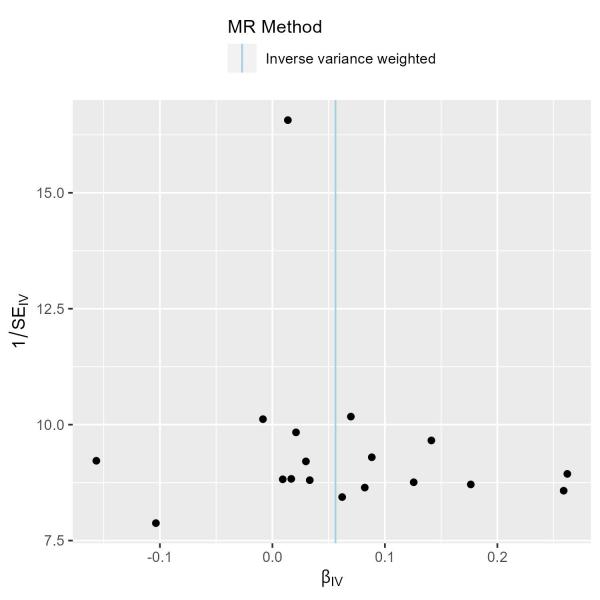

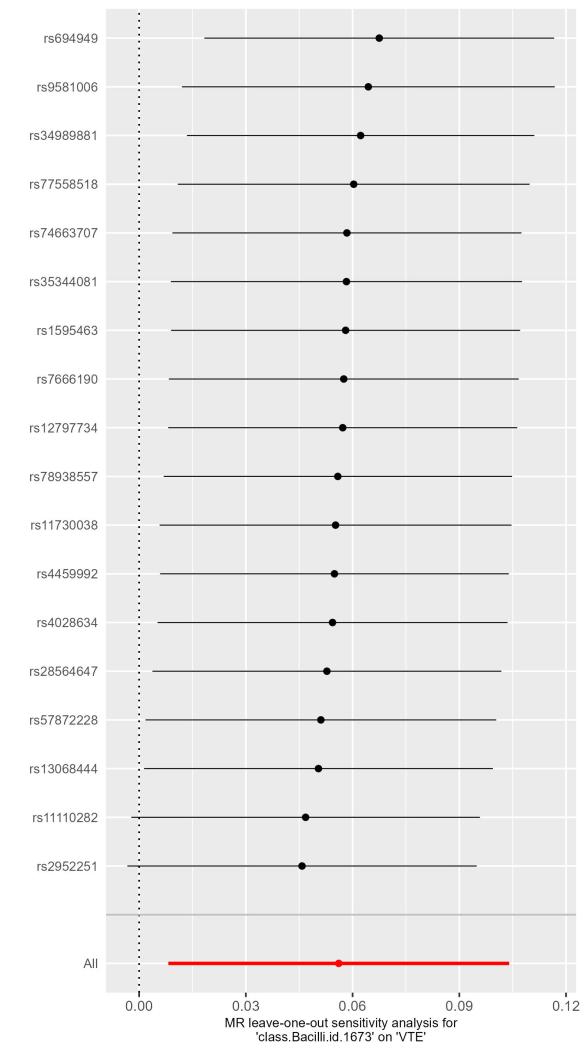

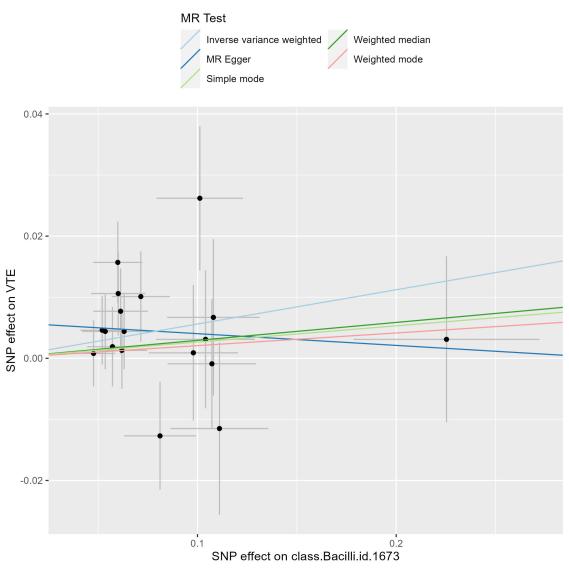


A

B

C

Supplement Figure 1. A. Leave-one out test plot of the causal effect of class.Bacilli.id.1673 on VTE risk; B. scatter plot of the causal effect of class.Bacilli.id.1673 on VTE risk; C.funnel plot of the causal effect of class.Bacilli.id.1673 on VTE risk.


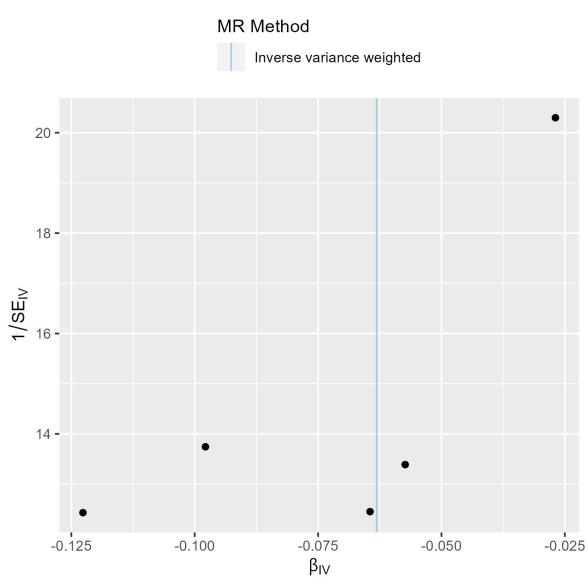

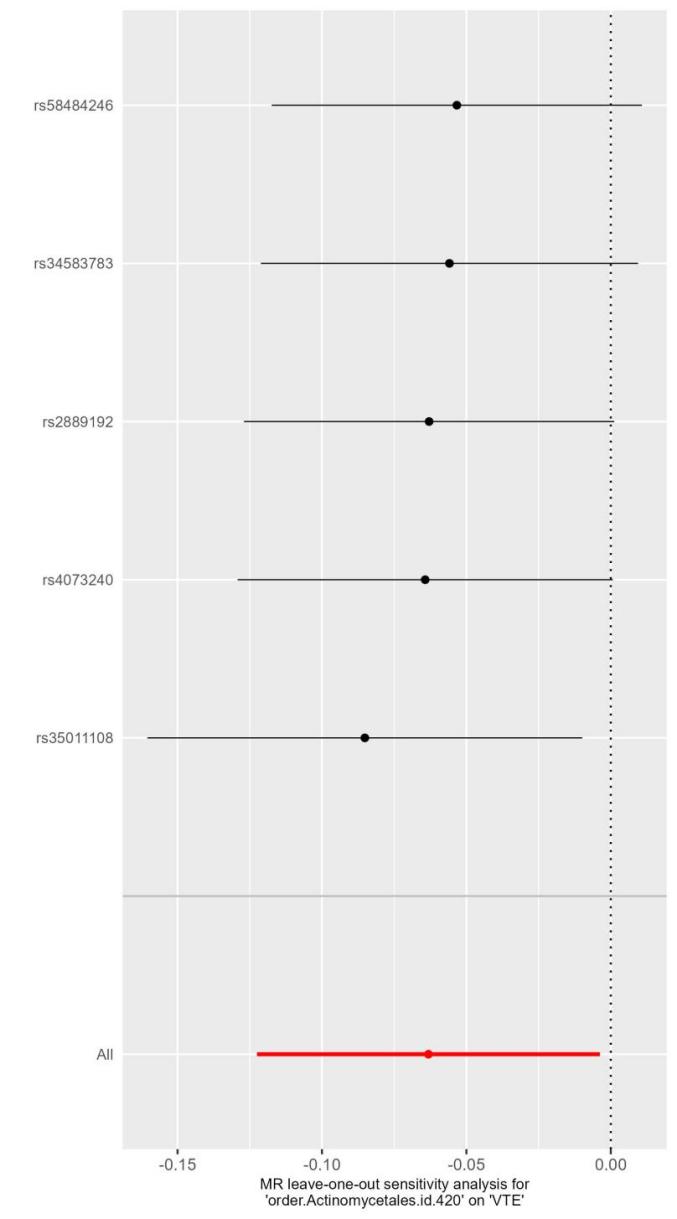

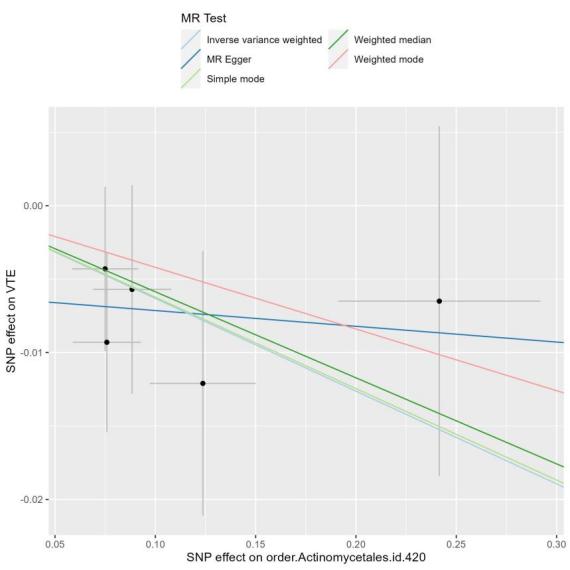


A

B

C

Supplement Figure 2. A. Leave-one out test plot of the causal effect of order.Actinomycetales.id.420 on VTE risk; B. scatter plot of the causal effect of order.Actinomycetales.id.420 on VTE risk; C. and funnel plot of the causal effect of order.Actinomycetales.id.420 on VTE risk.


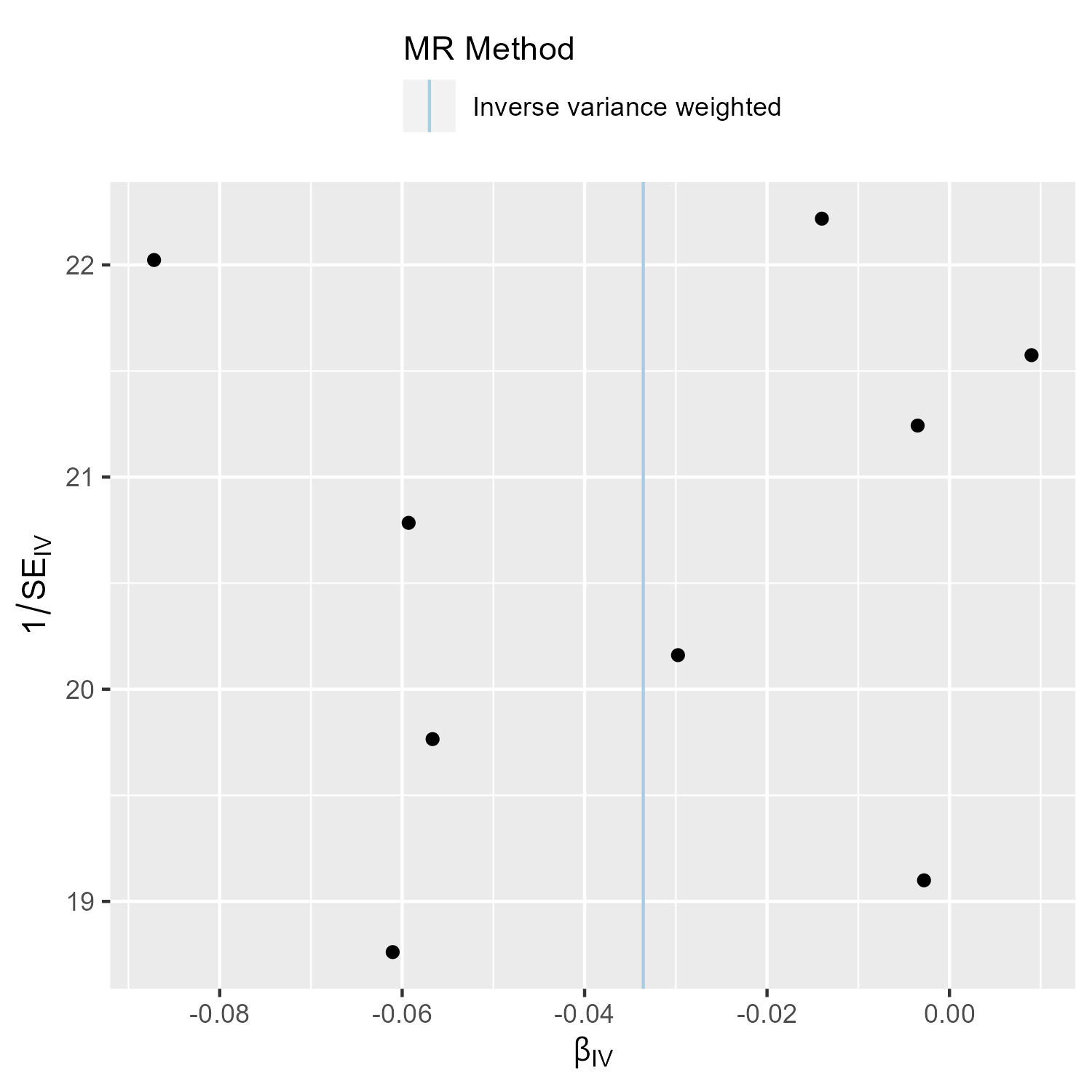

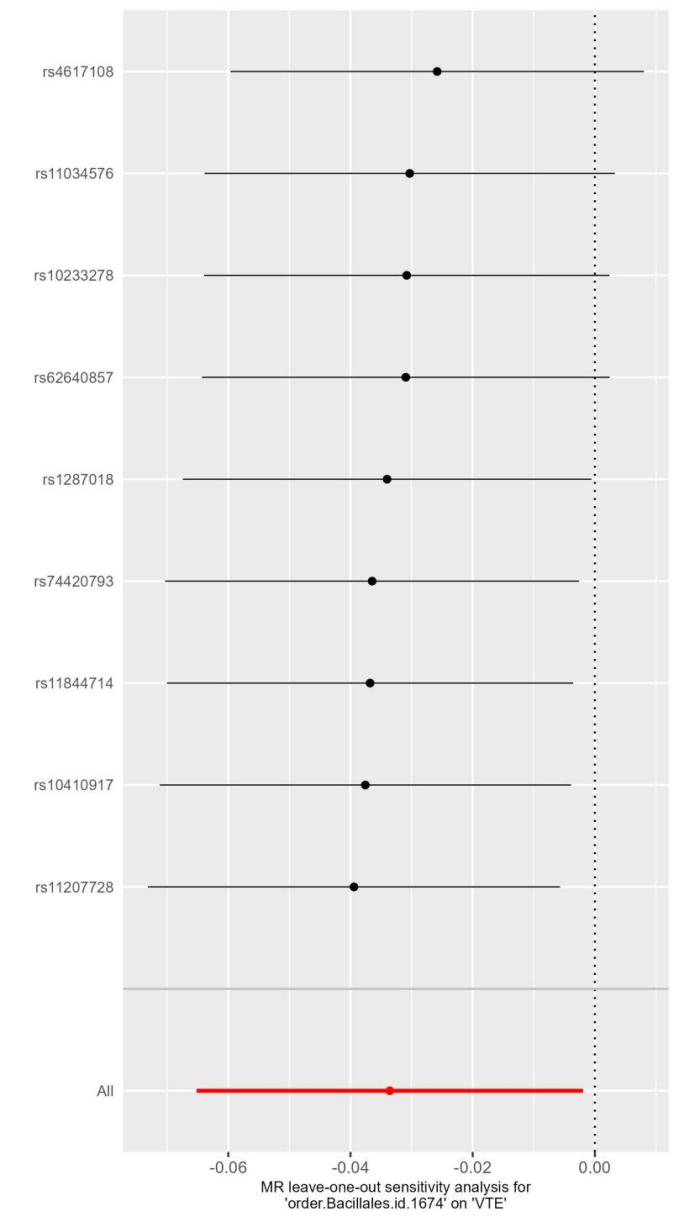

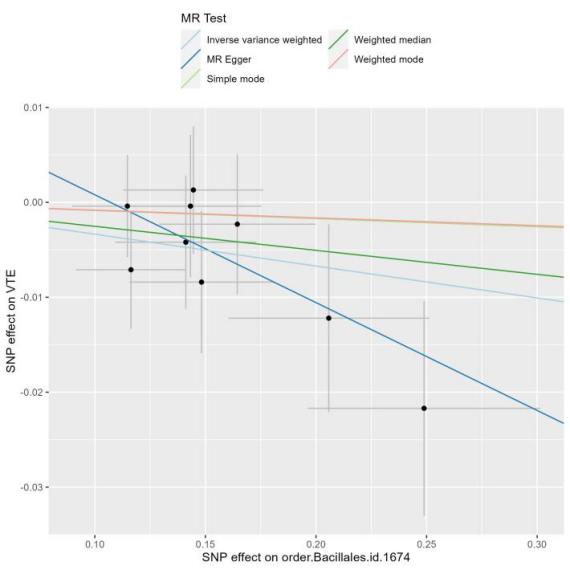


A

B

C


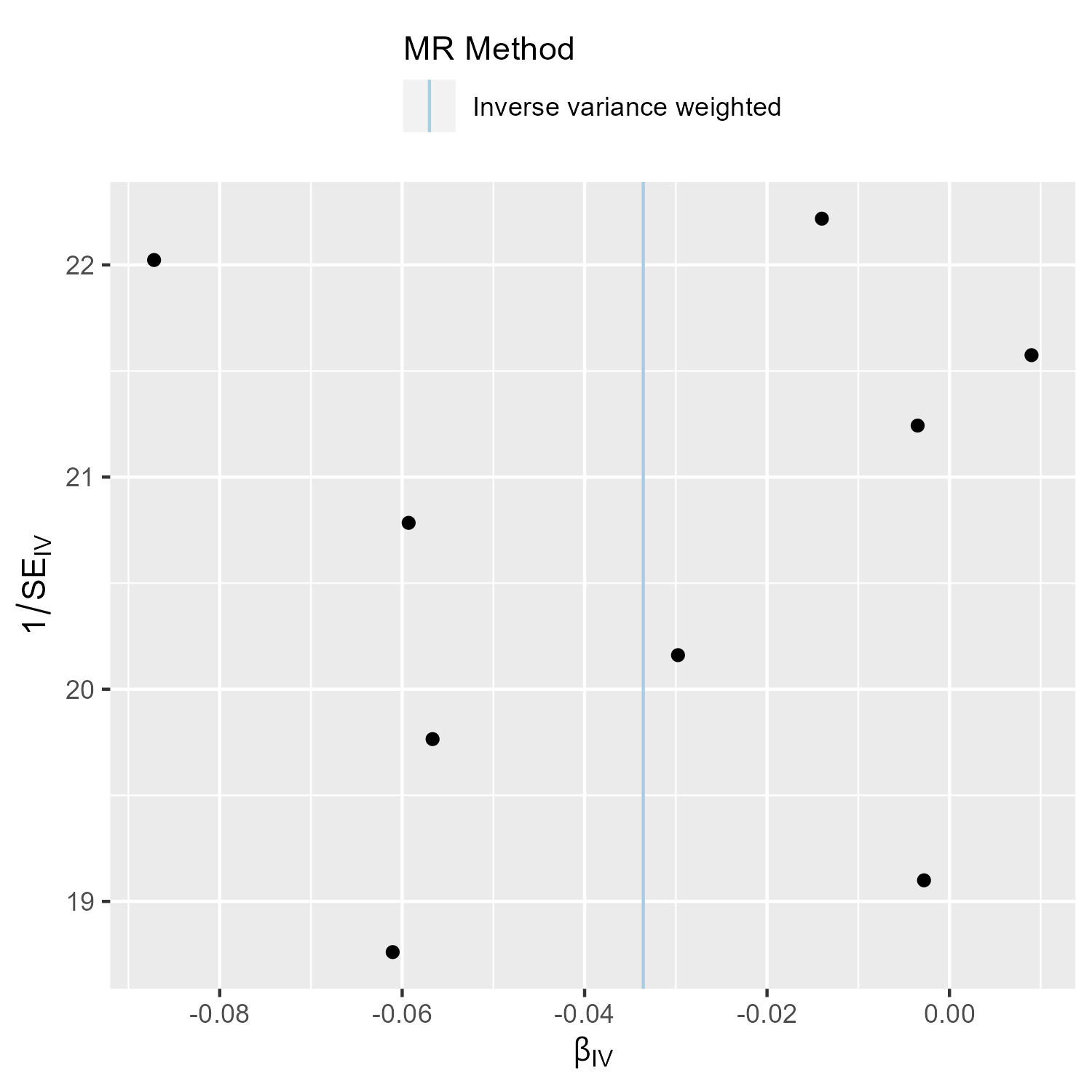


Supplement Figure 3. A. Leave-one out test plot of the causal effect of order.Bacillales.id.1674 on VTE risk; B. scatter plot of the causal effect of order.Bacillales.id.1674 on VTE risk; C. and funnel plot of the causal effect of order.Bacillales.id.1674 on VTE risk.


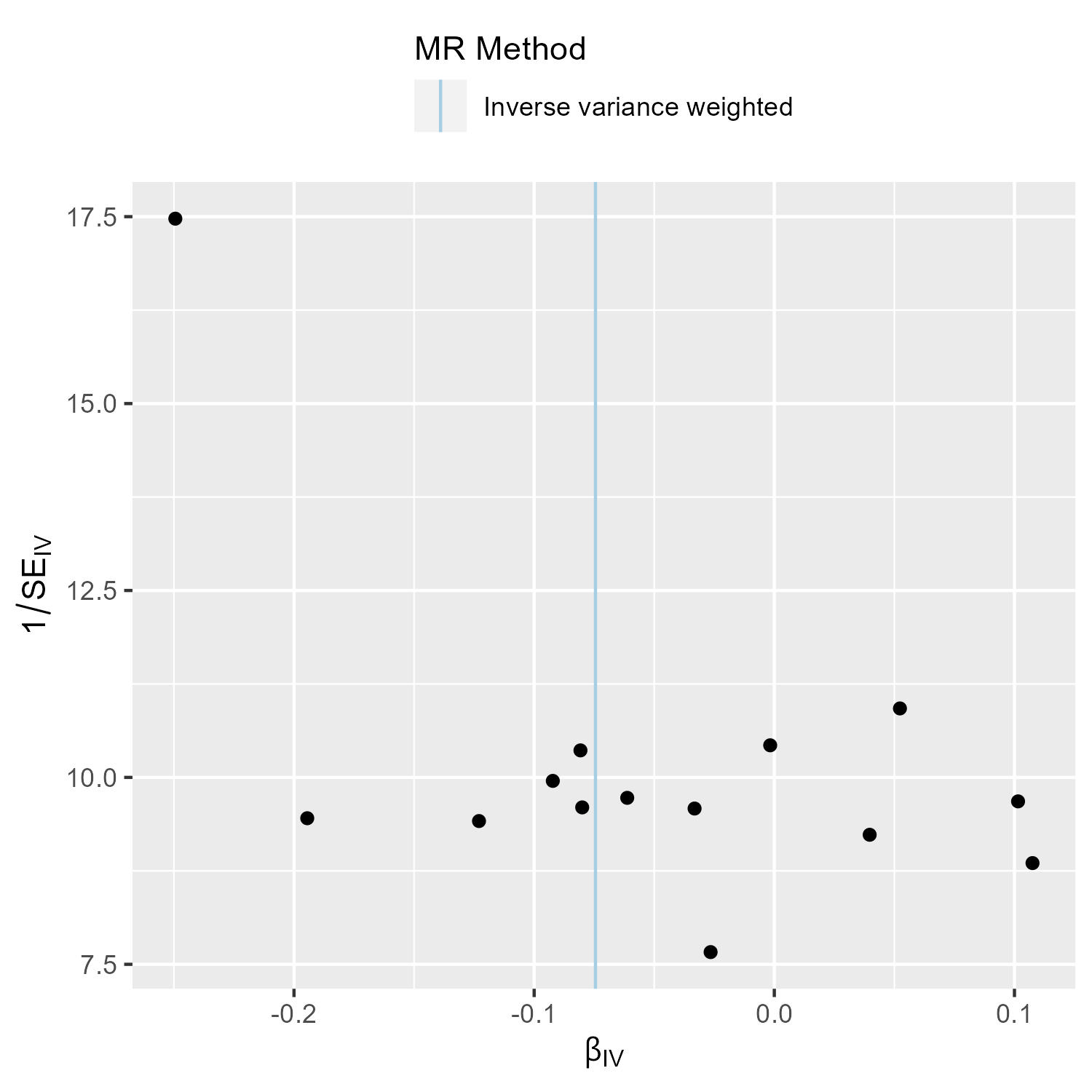

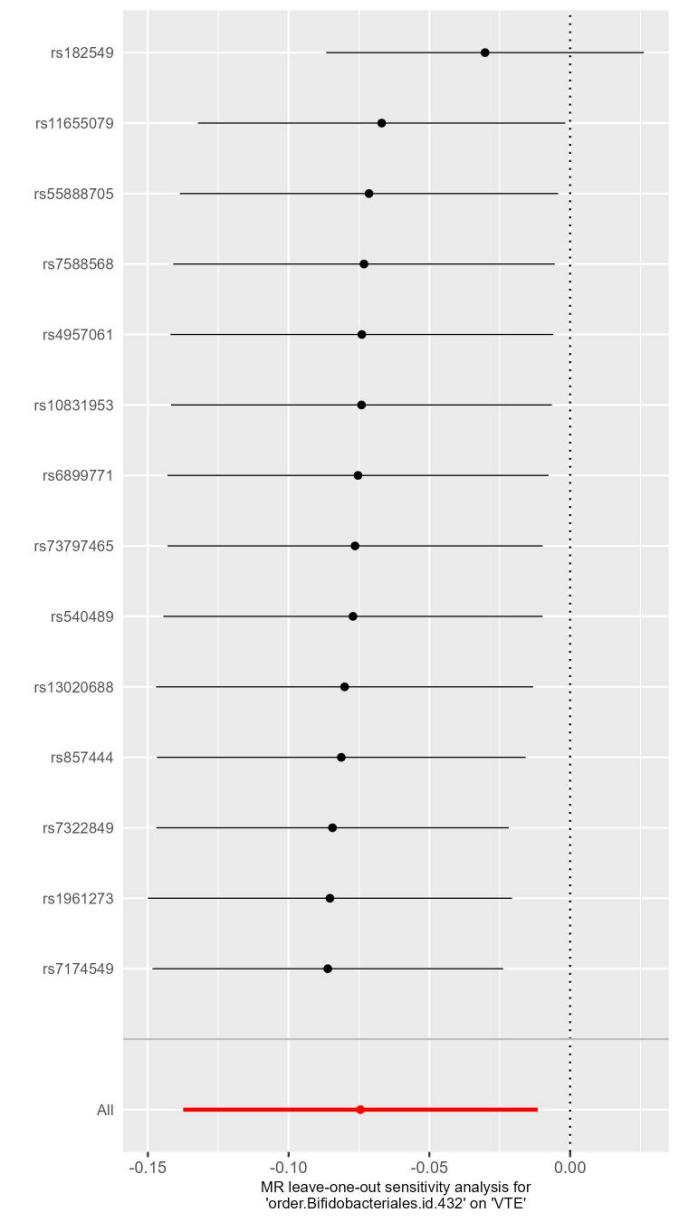

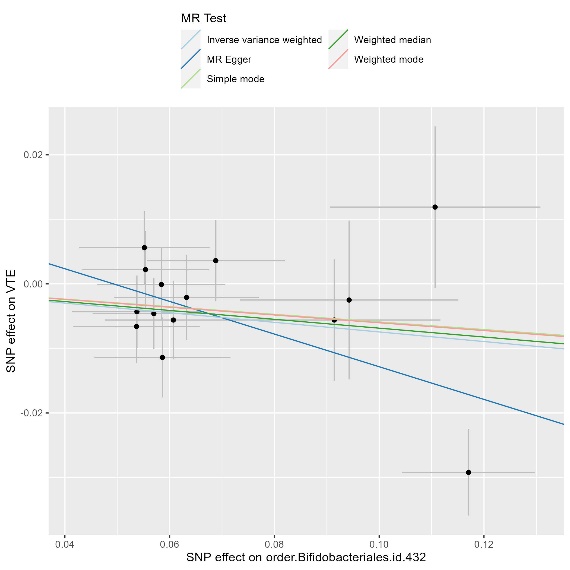


A

B

C

Supplement Figure 4. A. Leave-one out test plot of the causal effect of order.Bifidobacteriales.id.432 on VTE risk; B. scatter plot of the causal effect of order.Bifidobacteriales.id.432 on VTE risk; C. and funnel plot of the causal effect of order.Bifidobacteriales.id.432 on VTE risk.


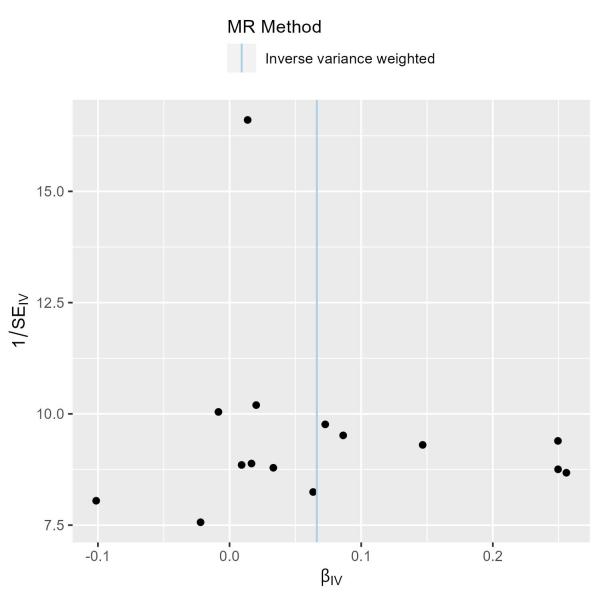

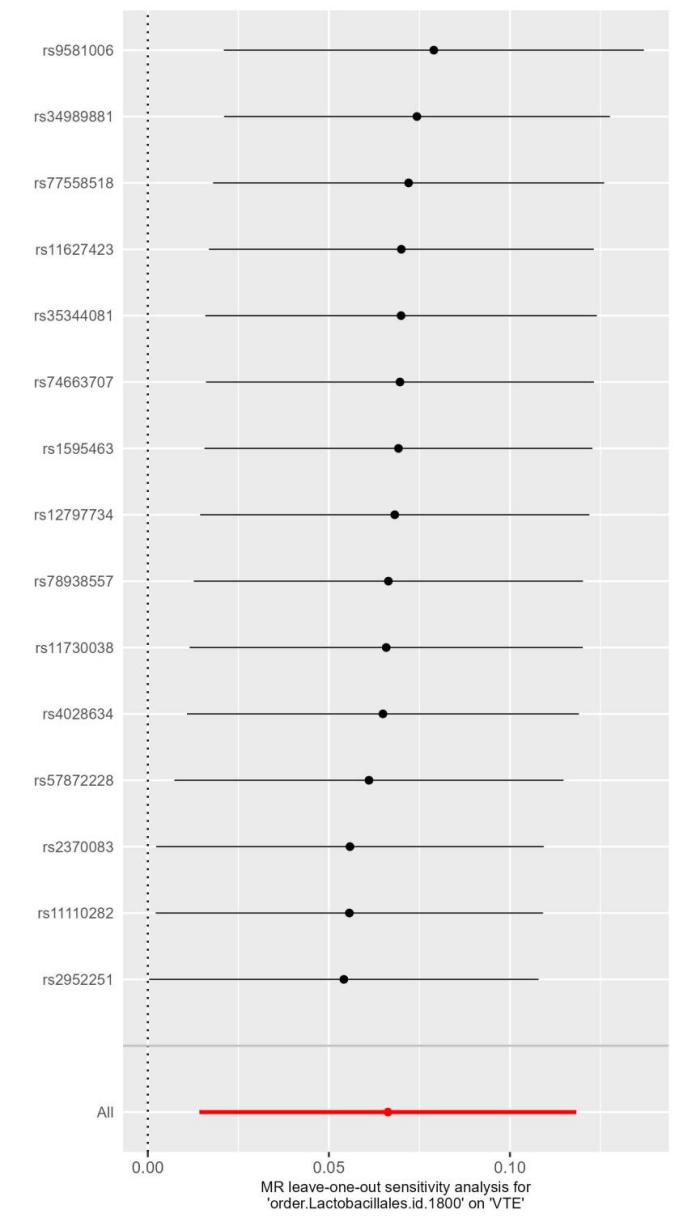

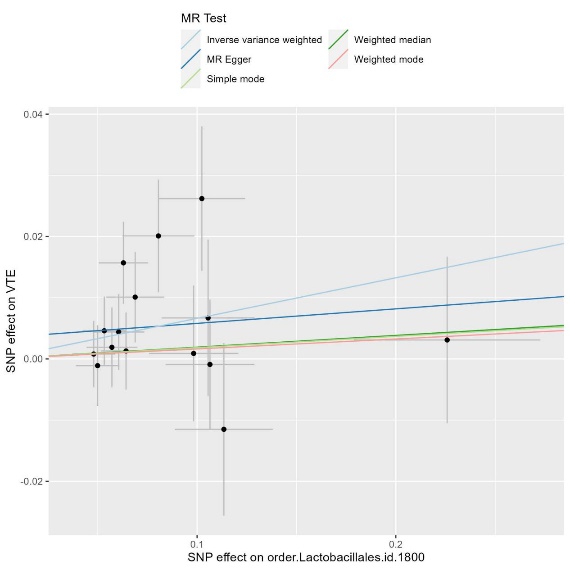


A

B

C

Supplement Figure 5. A. Leave-one out test plot of the causal effect of order.Lactobacillales.id.1800 on VTE risk; B. scatter plot of the causal effect of order.Lactobacillales.id.1800 on VTE risk; C. and funnel plot of the causal effect of order.Lactobacillales.id.1800 on VTE risk.


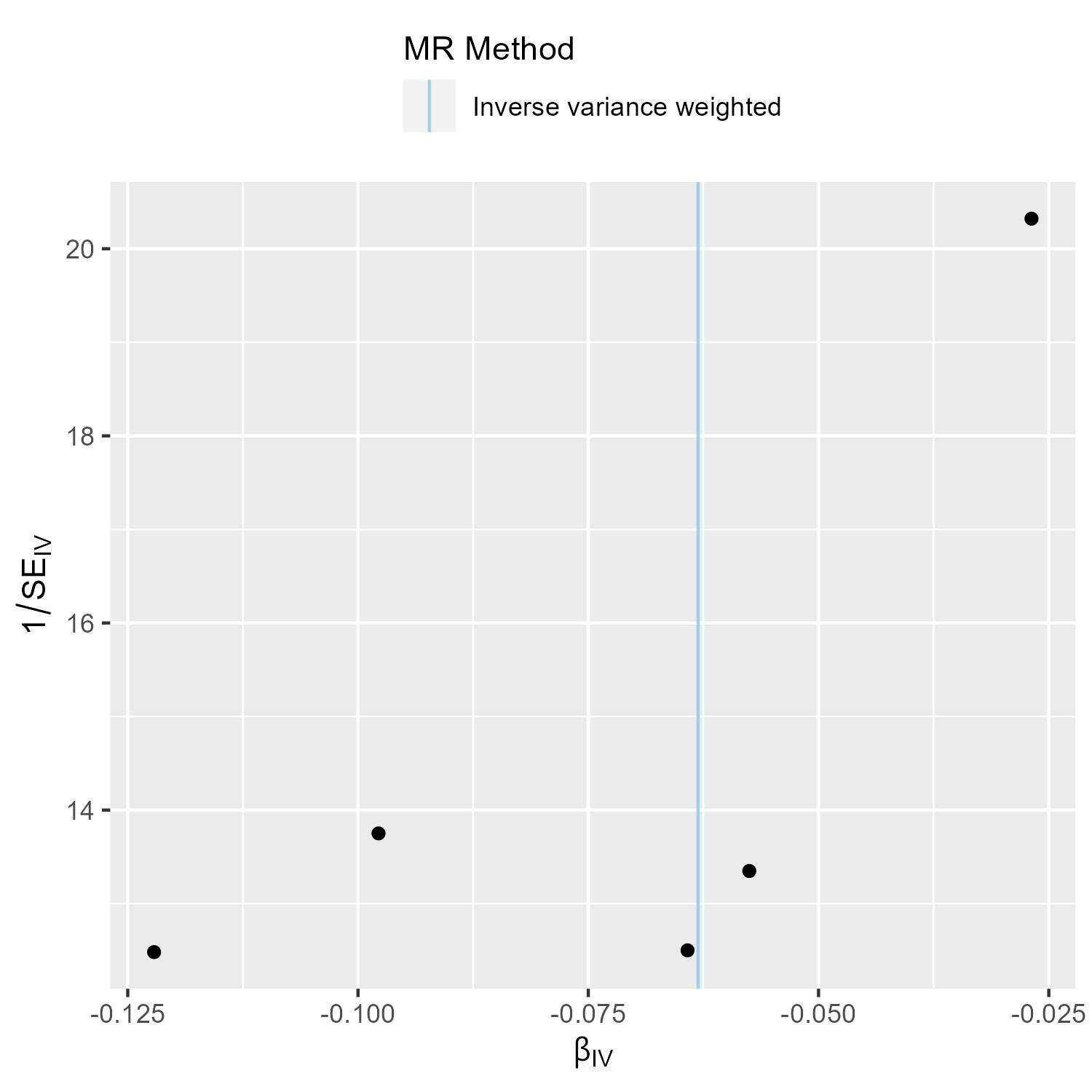

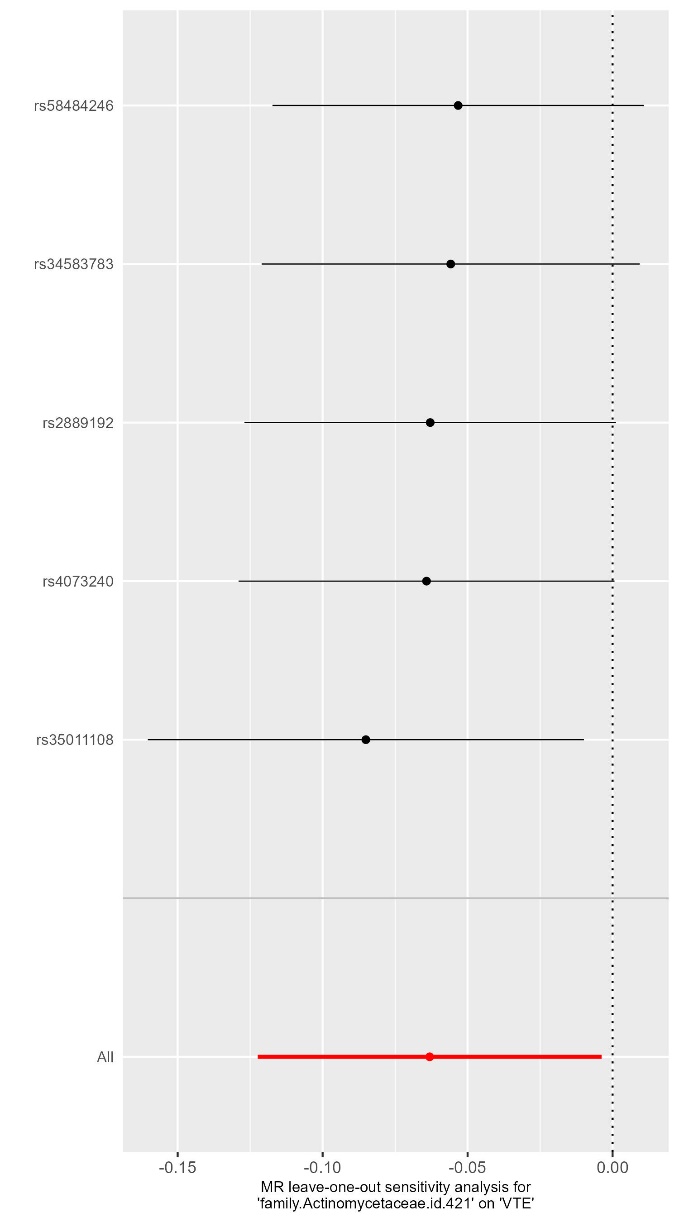

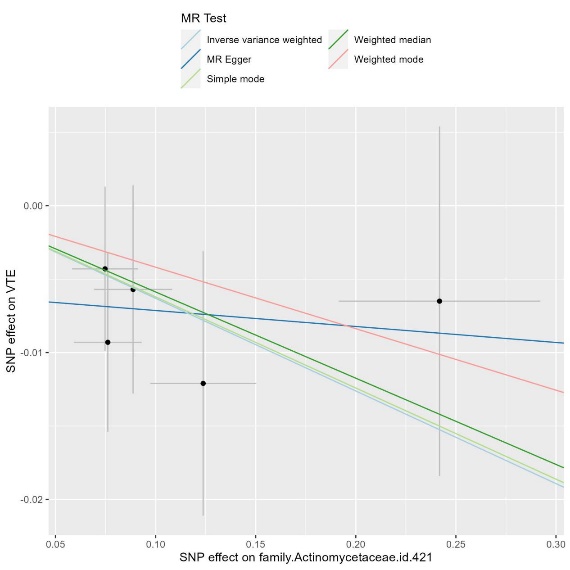


A

B

C

Supplement Figure 6. A. Leave-one out test plot of the causal effect of family.Actinomycetaceae.id.421 on VTE risk; B. scatter plot of the causal effect of family.Actinomycetaceae.id.421 on VTE risk; C. and funnel plot of the causal effect of family.Actinomycetaceae.id.421 on VTE risk.


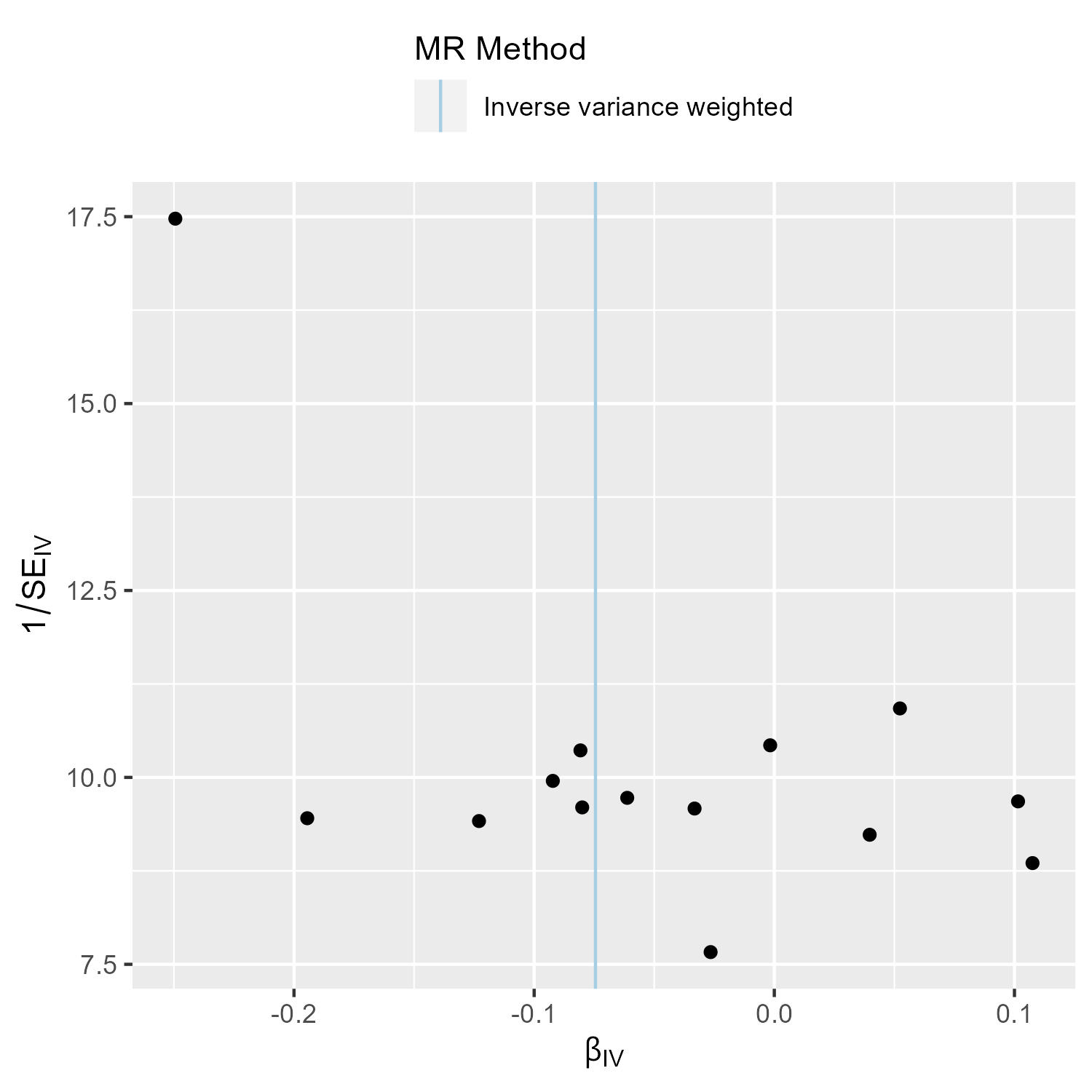

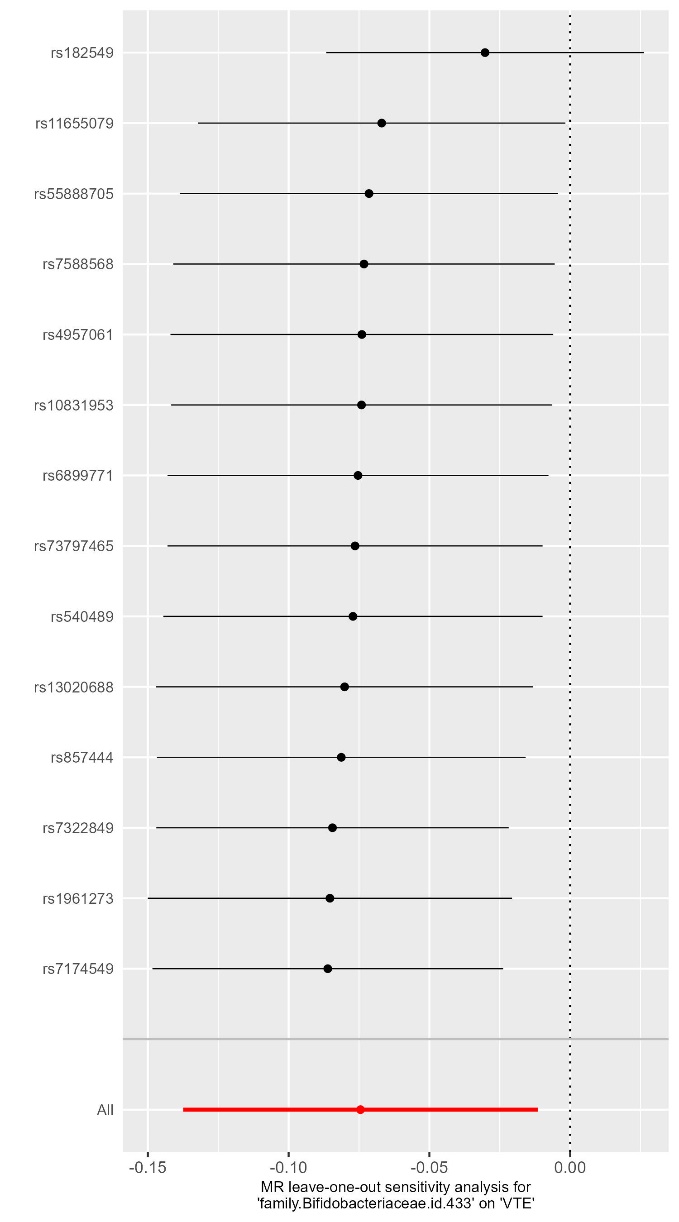

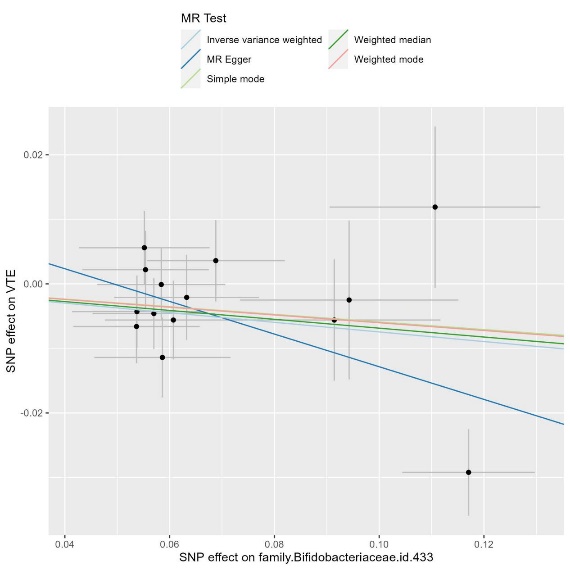


A

B

C

Supplement Figure 7. A. Leave-one out test plot of the causal effect of family.Bifidobacteriaceae.id.433 on VTE risk; B. scatter plot of the causal effect of family.Bifidobacteriaceae.id.433 on VTE risk; C. and funnel plot of the causal effect of family.Bifidobacteriaceae.id.433 on VTE risk.


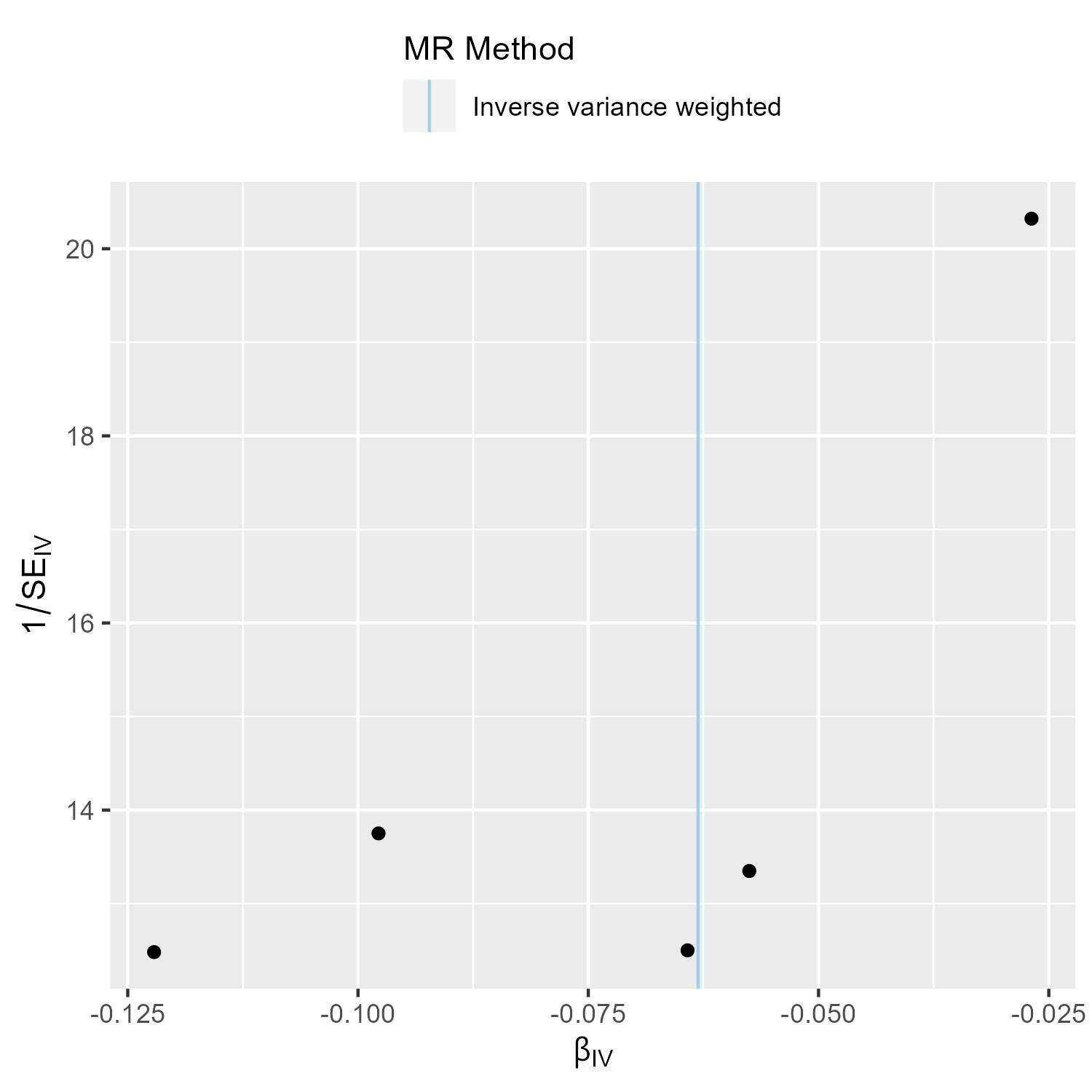

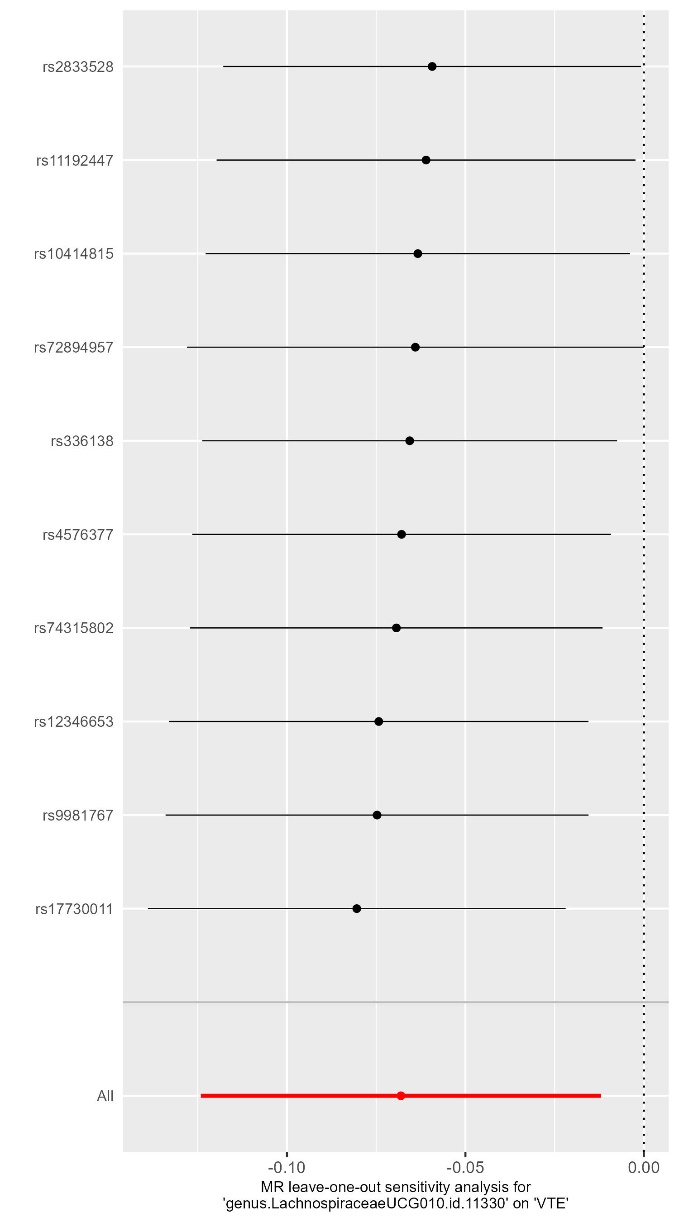

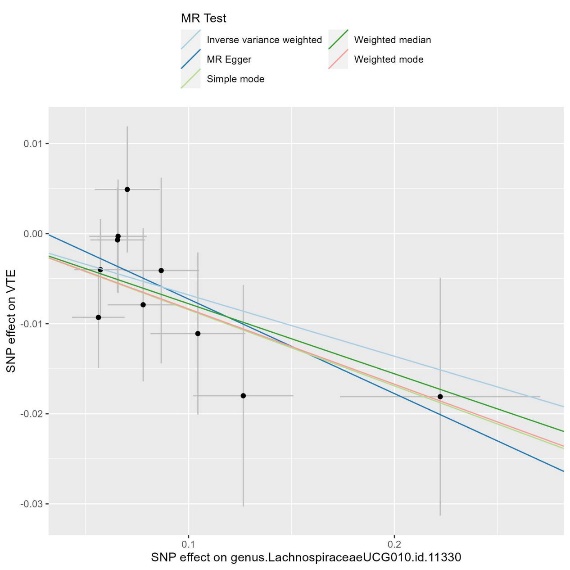


A

B

C

Supplement Figure 8. A. Leave-one out test plot of the causal effect of genus.LachnospiraceaeUCG010.id.11330 on VTE risk; B. scatter plot of the causal effect of genus.LachnospiraceaeUCG010.id.11330 on VTE risk; C. and funnel plot of the causal effect of genus.LachnospiraceaeUCG010.id.11330 on VTE risk.


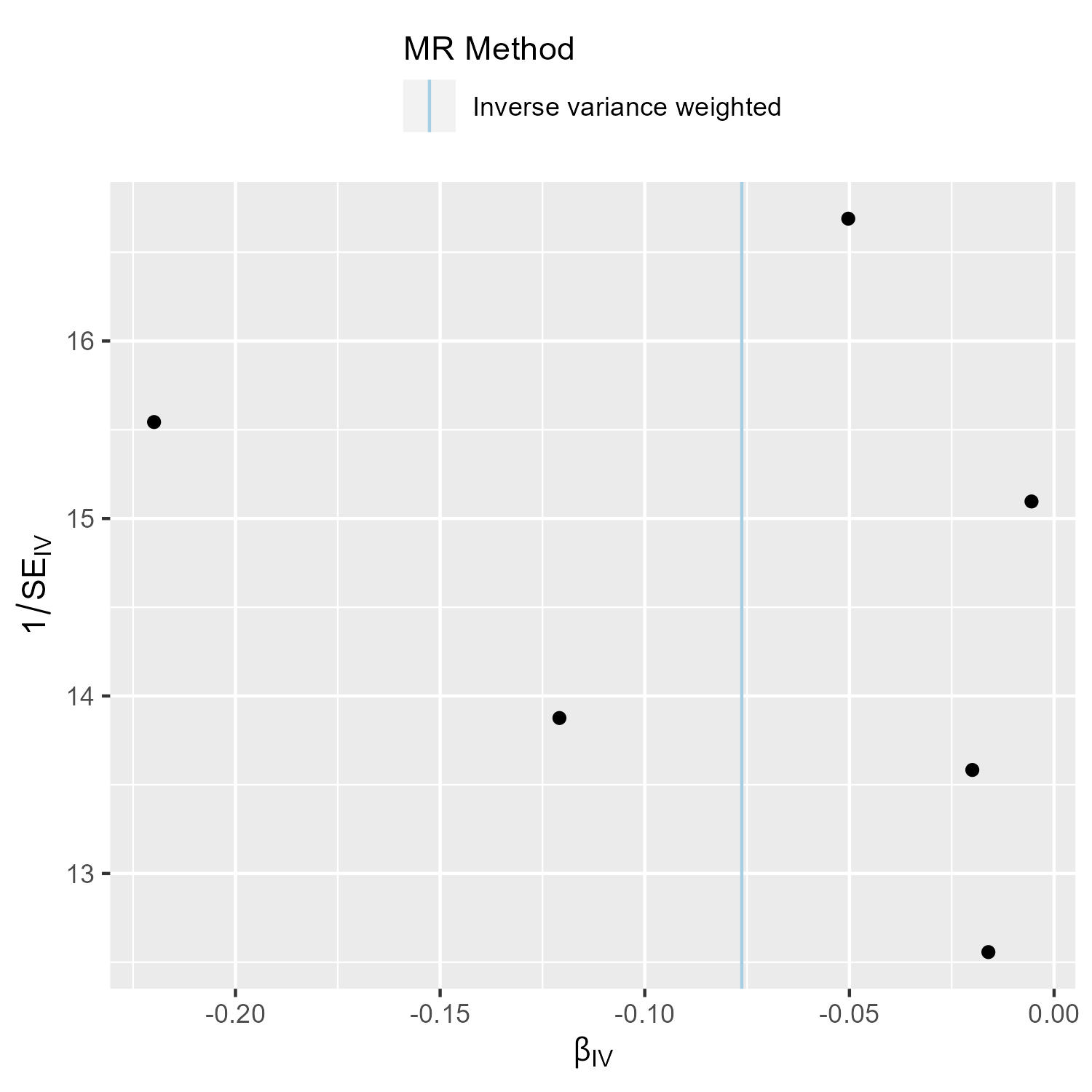

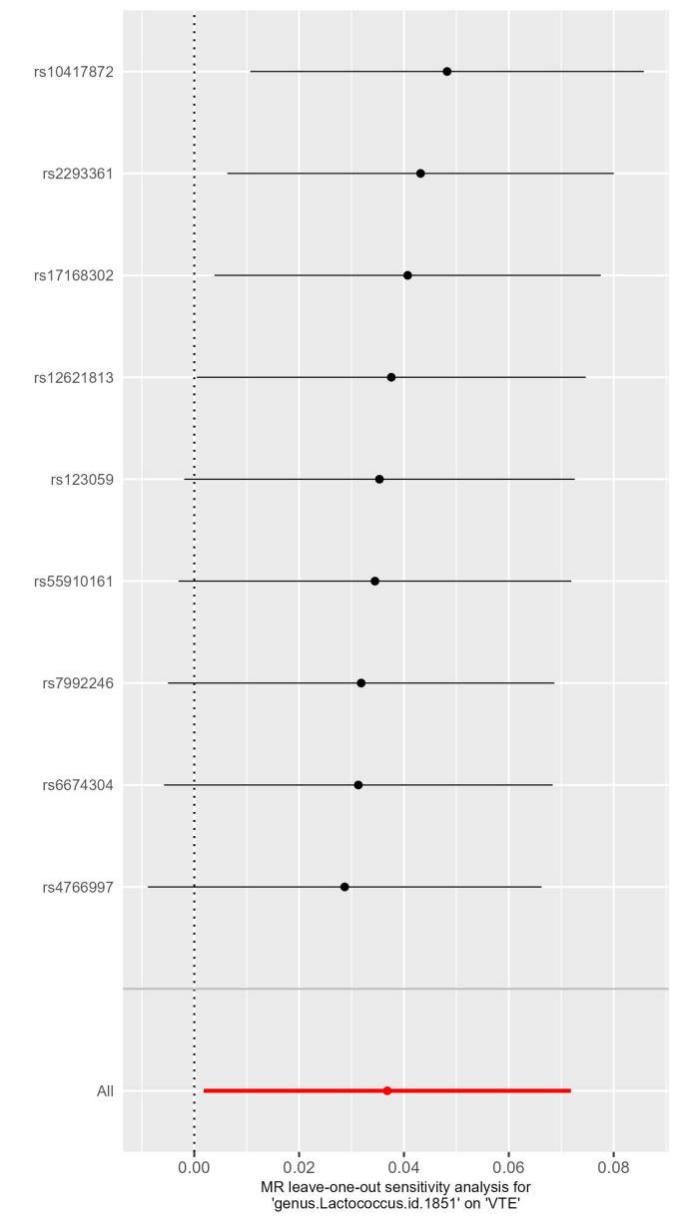

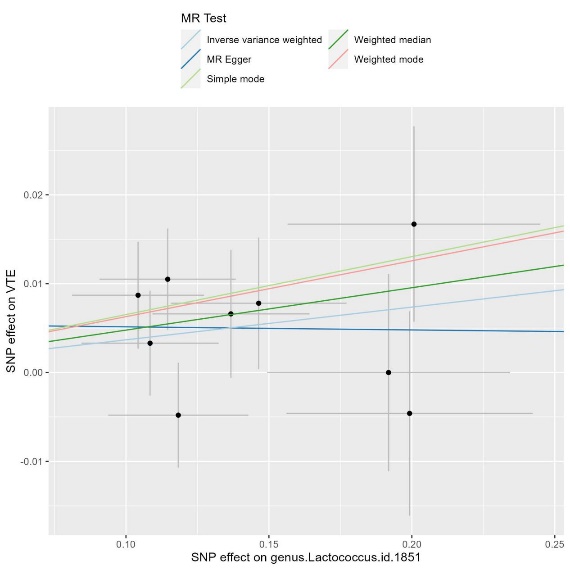


A

B

C

Supplement Figure 9. A. Leave-one out test plot of the causal effect of genus.Lactococcus.id.1851 on VTE risk; B. scatter plot of the causal effect of genus.Lactococcus.id.1851 on VTE risk ; C. and funnel plot of the causal effect of genus.Lactococcus.id.1851 on VTE risk.


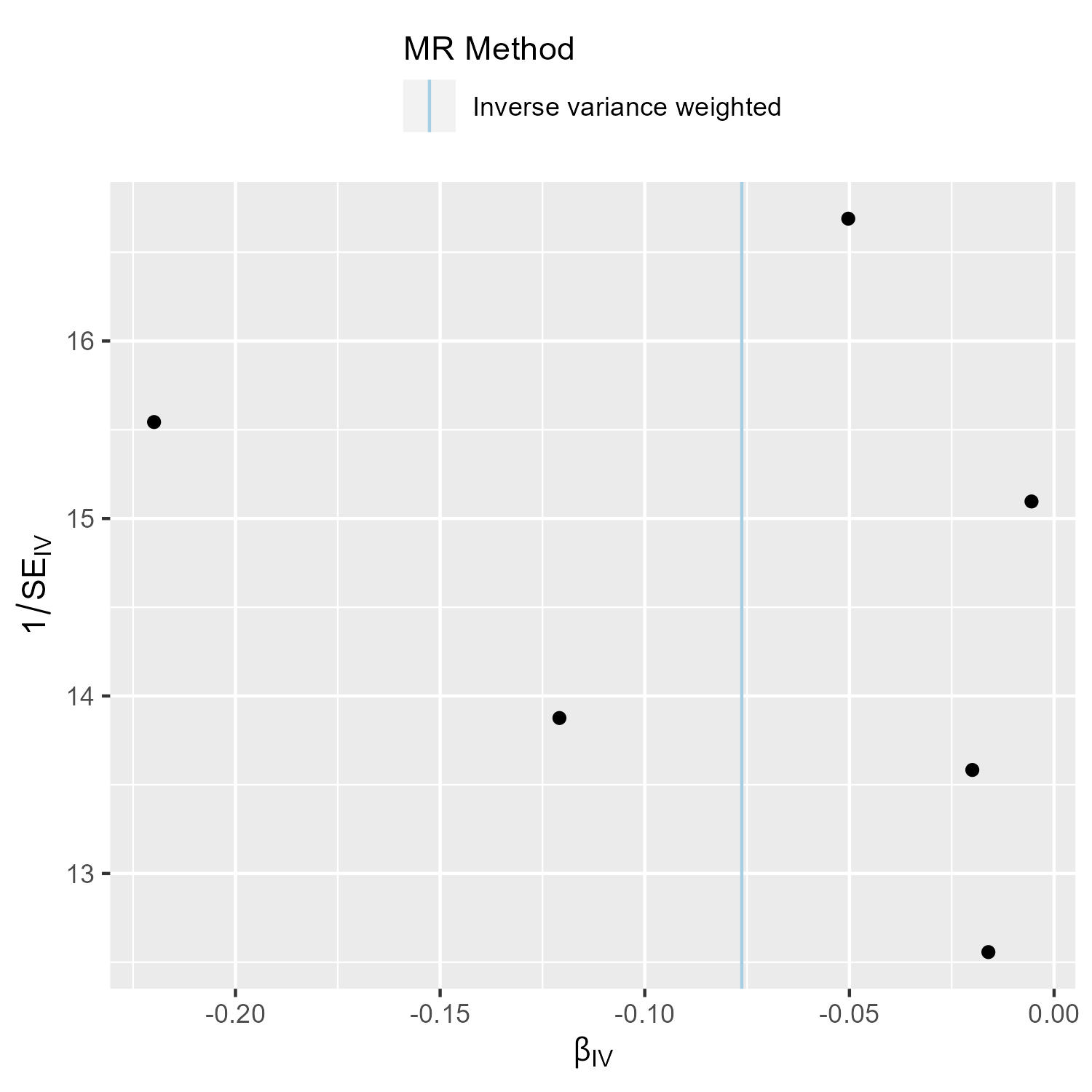

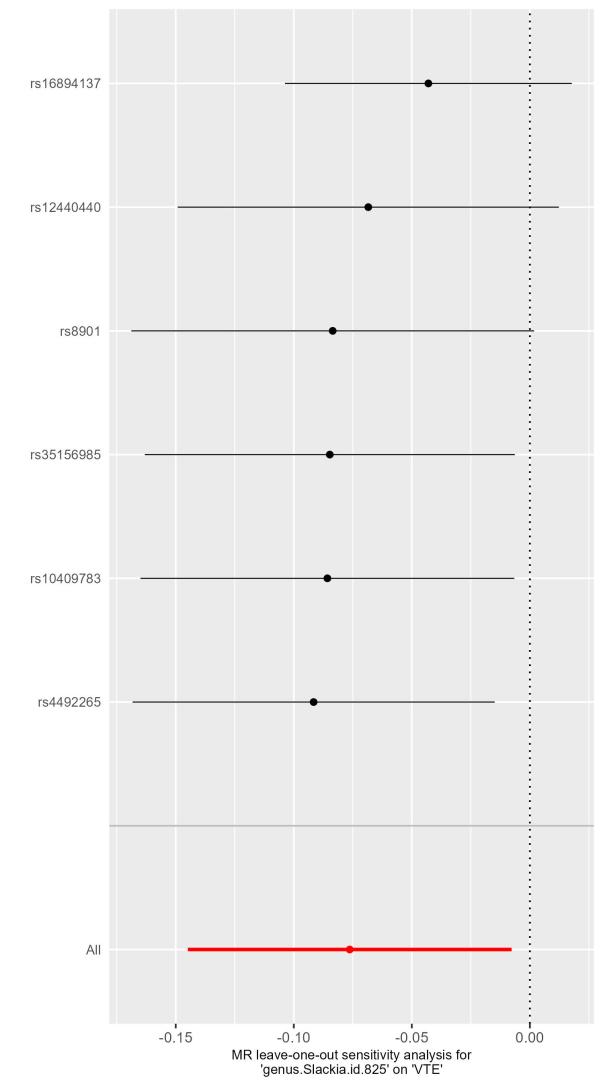

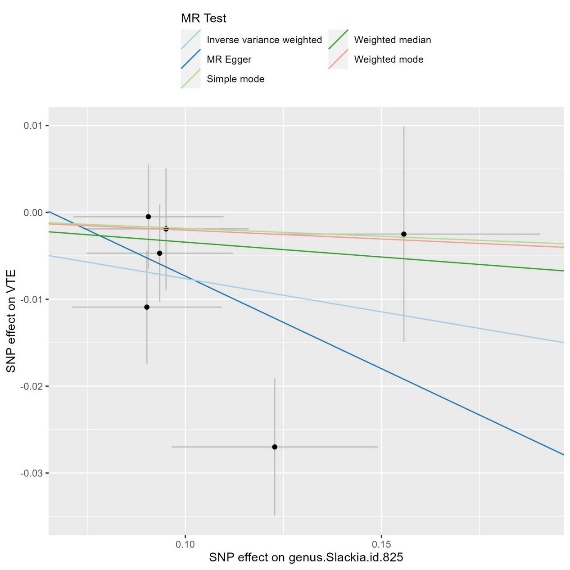


A

B

C

Supplement Figure 10. A. Leave-one out test plot of the causal effect of genus.Slackia.id.825 on VTE risk; B. scatter plot of the causal effect of genus.Slackia.id.825 on VTE risk; C. and funnel plotof the causal effect of genus.Slackia.id.825 on VTE risk.
